# The associations of mobile touch screen device use with musculoskeletal symptoms and exposures: A systematic review

**DOI:** 10.1371/journal.pone.0181220

**Published:** 2017-08-07

**Authors:** Siao Hui Toh, Pieter Coenen, Erin K. Howie, Leon M. Straker

**Affiliations:** 1 School of Physiotherapy and Exercise Science, Curtin University, Perth, Australia; 2 Physiotherapy Department, KK Women’s and Children’s Hospital, Singapore, Singapore; 3 Department of Public and Occupational Health, EMGO+ Institute for Health and Care Research, VU University Medical Centre, Amsterdam, the Netherlands; 4 Department of Health, Human Performance and Recreation, University of Arkansas, Fayetteville, Arkansas, United States of America; Bern University of Applied Science, SWITZERLAND

## Abstract

**Background:**

The use of mobile touch screen devices (MTSDs) has increased rapidly over the last decade, and there are concerns that their use may have negative musculoskeletal consequences; yet evidence on the association of MTSD use with musculoskeletal symptoms and exposures is currently dispersed. The aim of this study was to systematically review available literature on musculoskeletal symptoms and exposures associated with MTSD use. The synthesised information may facilitate wise use of MTSDs and may identify areas in need of further research.

**Methods:**

EMBASE, Medline, Scopus, PsycINFO and Proquest electronic databases were searched for articles published up to June 2016, using keywords describing MTSD, musculoskeletal symptoms (e.g. pain, discomfort) and musculoskeletal exposures (e.g. posture, muscle activity). Two reviewers independently screened the articles, extracted relevant data and assessed methodological quality of included studies. Due to heterogeneity in the studies, a meta-analysis was not possible and a structured narrative synthesis of the findings was undertaken.

**Results:**

A total of 9,908 articles were screened for eligibility with 45 articles finally included for review. Included articles were of cross-sectional, case-control or experimental laboratory study designs. No longitudinal studies were identified. Findings were presented and discussed in terms of the amount, features, tasks and positions of MTSD use and its association with musculoskeletal symptoms and musculoskeletal exposures.

**Conclusions:**

There is limited evidence that MTSD use, and various aspects of its use (i.e. amount of usage, features, tasks and positions) are associated with musculoskeletal symptoms and exposures. This is due to mainly low quality experimental and case-control laboratory studies, with few cross-sectional and no longitudinal studies. Further research is warranted in order to develop guidelines for wise use of MTSDs.

## 1 Introduction

Mobile touch screen devices (MTSDs) are portable devices with a touch screen interface that can be used with stylus or finger touch, such as smartphones and tablet computers. There has been an increase in ownership and usage of MTSDs among adults and children over the last decade [1, 2]. Recent figures from 2016 showed that the majority of teenagers aged 14 to 18 years in the USA (87%) [3] and 12 to 15 years in the UK (79%) reported owning a smartphone [4]. Smartphone ownership is even higher among adults, with 92% and 95% of the adults aged 18 to 34 years in the USA and Australia respectively reported to own a smartphone [2]. Another recent survey in the USA showed that in 2015 adult users spent approximately three hours daily (excluding voice activities) on their mobile devices, which was double the duration spent in 2012 [5]. The substantial ownership and usage of MTSDs is expected to continue to increase in the years ahead [5], and is therefore an important societal change.

Apart from potential social, mental and behavioural effects such as negative impacts on social relationships, depression and sleep quality [6, 7], the increased MTSD use has also raised concerns for potential physical health effects including musculoskeletal symptoms [8, 9]. The use of more traditional electronic devices, i.e. desktop and laptop computers, has been associated with musculoskeletal symptoms in several epidemiological studies [10–13]. Laboratory research has demonstrated that musculoskeletal exposures including awkward postures, lack of posture variation, and altered muscle activity are associated with computer use and these may explain the development of musculoskeletal symptoms due to the use of traditional devices [14, 15]. These indications of changes in postures and muscle activity thus provide support for concerns about the potential risk of symptoms from MTSD use.

However, the use of MTSDs is different from traditional electronic devices such as desktop or laptop computers or physical keypad phones, due to their portability and control interaction via a touch screen interface rather than via a keyboard and/or mouse. MTSDs may therefore be associated with different musculoskeletal exposures (e.g. postures or muscle activity), which may create different risks for musculoskeletal symptoms than traditional devices. Due to their portability, MTSDs may be used in various non-traditional workstations and postures (e.g. on a sofa or while using public transport), which may be associated with different musculoskeletal exposures than while using a device whilst seated at a desk [16]. Due to their designs that do not allow wrist and fingers to rest on the screen surface, the use of a touch screen may incur further exposures to awkward neck/shoulder postures and distal upper extremity muscles [17, 18]. As a result of this, it has been reported that tablet use may cause higher musculoskeletal stresses on the neck compared to desktop computer use [19], and higher stress on the wrist during smartphone use compared to when using a keypad phone [20]. Moreover, higher activity in the neck/shoulder muscles [19, 21], and lower activity of the wrist extensor muscles [20, 22] have been reported during MTSD use compared to when using traditional electronic devices. The different musculoskeletal implications from the use of MTSDs versus traditional devices were also reinforced by the few cross-sectional studies that have found evidence for an association between MTSD use and musculoskeletal symptoms [23–25].

These findings support concerns about MTSD use posing a risk for musculoskeletal symptoms. Information on the associations between musculoskeletal symptoms, musculoskeletal exposures and MTSD use is therefore important in understanding the potential musculoskeletal implications associated with the use of these devices. Such evidence is currently dispersed. Although there are reports of studies investigating different aspects of MTSD use, to the best of our knowledge, a systematic review of the current evidence on musculoskeletal symptoms and exposures associated with MTSD use is not yet available. Therefore, the aim of this study was to systematically review available literature on musculoskeletal symptoms and exposures associated with the use of MTSDs. The synthesised information may inform guidelines for wise use of MTSDs and identify areas in need of further research.

## 2 Methods

### 2.1 Search strategy

The protocol for this systematic review was registered in PROSPERO [26]. Systematic searches of the literature were conducted in five electronic databases (EMBASE, Medline, Scopus, PsycINFO and Proquest) up to June 2016. The first touch screen phone (Simon; IBM) and the first personal digital assistant (Newton; Apple) were made available in 1993 [27], hence the search was conducted for relevant studies published from 1993 onwards. Keywords describing MTSD (e.g. smart phone, tablet computer, touch screen, mobile device), musculoskeletal symptoms (e.g. pain, musculoskeletal pain, discomfort) and musculoskeletal exposures (e.g. posture, muscle activity, electromyography) were used (see S1 File).

Two reviewers (SHT and PC) independently screened all potential titles, abstracts, and if needed, full-texts for eligibility. Disagreement for inclusion was resolved through a consensus meeting or consulting a third reviewer (EKH). Studies were included if: (1) the study examined the use of MTSD and associated musculoskeletal outcomes (i.e., musculoskeletal symptoms such as discomfort or pain, and/or musculoskeletal exposures such as postures or muscle activity); (2) the study described original research from laboratory or field studies or cross-sectional or longitudinal epidemiological studies (i.e., excluding case reports, reviews, conference proceedings, editorials and letters) written in English. Studies that described the use of MTSD as interventions for managing health conditions (e.g. text message or mobile applications for telemedicine) or examined outcomes of gait or balance parameters were excluded. Reference lists of all included full-text articles were screened for additional papers. Authors of articles were contacted to seek clarification where insufficient information was provided.

### 2.2 Data extraction and methodological quality assessment

In line with the PROSPERO registration, due to the heterogeneity in study designs, methods, outcomes and data presented in the included articles, a structured narrative synthesis was undertaken as the planned meta-analysis was not able to be carried out. For the narrative synthesis, relevant data from all included articles were extracted and methodological quality was assessed by two reviewers (SH and PC) independently. Disagreement between the two reviewers was resolved through a consensus meeting or consulting a third reviewer (EKH). Findings of the articles were described and synthesized in a narrative format.

Data for the following categories were extracted from the included studies: study purpose, study design, study population, musculoskeletal symptoms (if applicable), musculoskeletal exposures and/or physiological responses (if applicable), statistical analyses and results. Other musculoskeletal outcomes which did not fall under musculoskeletal symptoms or exposures, such as pressure pain threshold or median nerve size, were identified in some of the studies and were extracted separately as physiological responses may provide further insights on musculoskeletal outcomes of MTSD use. As various terminologies of head, neck and gaze angles were present among the studies, terminologies defined by Straker and colleagues [28] were used to enable consistent comparison between studies.

A criteria list adapted from existing methodological quality assessment scales [29–32] was used (see S2 File), with criteria on study purpose, study design, study population, musculoskeletal symptoms, musculoskeletal exposures and/or physiological responses, statistical analyses and results. Each of these categories were scored positive (+), negative (-), unclear (?, i.e. insufficient information available) or not applicable (NA). For each study, the percentage of positive categories scored was tabulated.

## 3 Results

### 3.1 Search results

**Fig 1:**
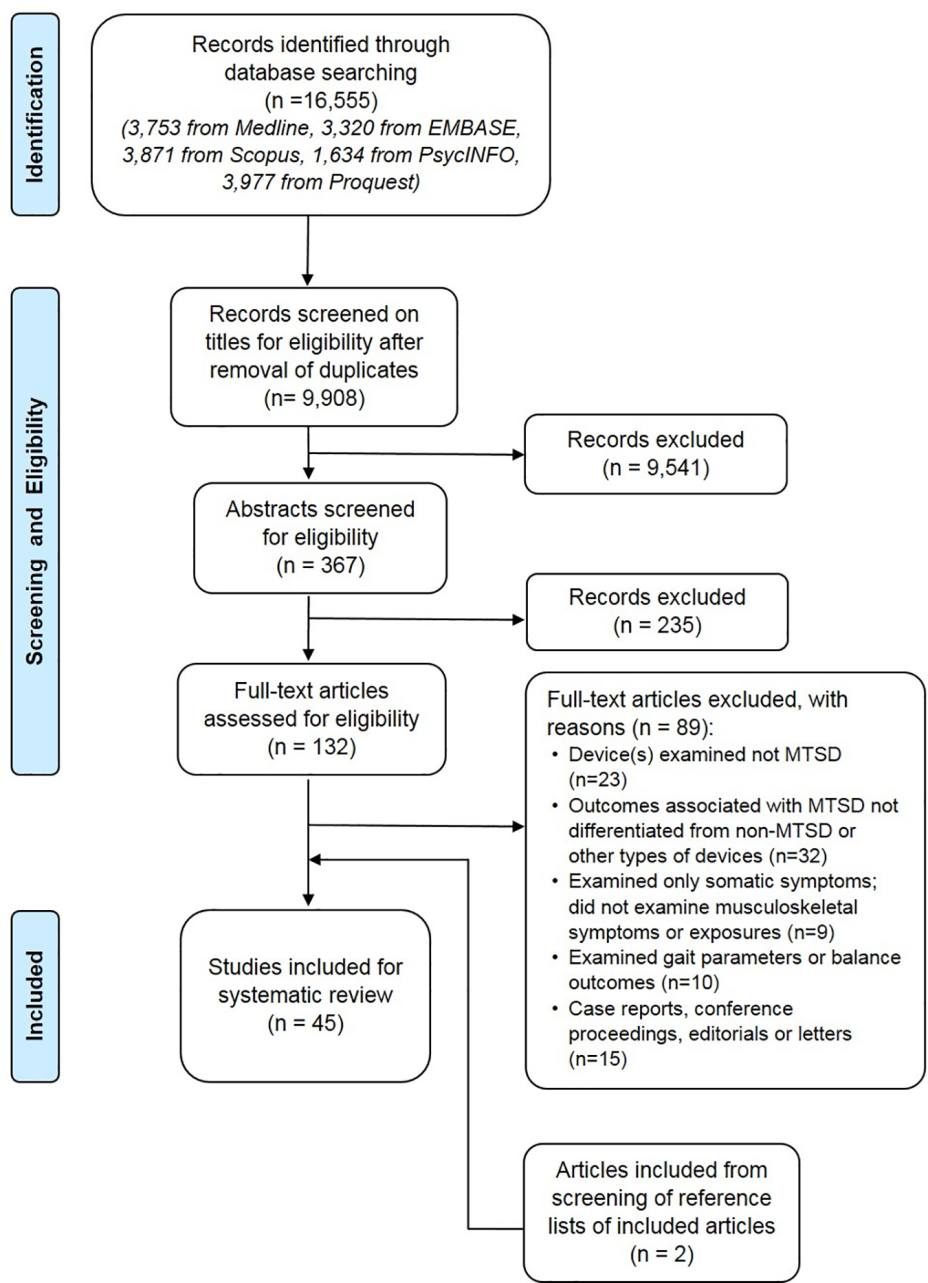
PRISMA flow chart for selecting relevant articles.

From the database search, after removing duplicates, 9,908 references were retrieved and screened for eligibility based on their titles (Fig 1). Following that, 367 abstracts and 132 full-text articles were assessed for eligibility. Articles were excluded due to: the device(s) being examined was not a MTSD, the outcomes associated with MTSDs were not being differentiated from non-MTSDs or other types of devices; or only somatic symptoms (e.g. headache, dizziness), gait or balance parameters were examined (not addressing musculoskeletal outcomes). Two additional articles were included after screening reference lists of the included articles. A total of 45 articles were included in this review.

### 3.2 Participants and study designs

Included articles were published from 2007 to 2016 and described assessments of participants with reported mean ages ranging from 5.8 to 67.5 years old. Forty articles reported studies conducted with adults, mostly young adults, four with young children or adolescents, while three articles did not report the age of participants. Included articles described six cross-sectional studies (see S3 File and S6 File), nine case-control laboratory studies (see S4 File, S7 File and S9 File) and 32 experimental laboratory studies (see S5 File, S8 File and S10 File), with two articles describing more than one type of study design [33, 34]. No longitudinal studies were identified.

Cross-sectional studies examined aspects of smartphone and tablet use e.g. duration and/or postures of use, with musculoskeletal symptoms and exposures using surveys or observational measurement methods. Experimental and case-control laboratory studies investigated: amount of usage, features (e.g. tilt angle, touch screen size, keyboard design, surface curvature), tasks (e.g. typing, gaming, internet browsing, reading, watching videos) and positions (e.g. handhold position, workstations such as desk, lap or sofa, and gross postures such as sitting or standing) of MTSD use with musculoskeletal symptoms, musculoskeletal exposures and physiological responses. For case-control laboratory studies, participants were categorised with respect to their symptoms (i.e., symptomatic or asymptomatic), amount of smartphone usage (i.e. more or less than four hours per day), and extent of “addiction” to smartphones (via questionnaires). Individual differences in participants, i.e. between symptomatic and asymptomatic, males and females or among different hand sizes and shapes, were also examined in a few of the studies.

### 3.3 Methodological quality

Included studies scored on average 55.7% on the methodological quality scale, ranging from 14.3% to 85.7% (Table 1). Slightly less than one third (n = 13/45) of all the included studies scored 50% and above. All the studies except one study [35] scored negative (-) or unclear (?) for musculoskeletal symptoms, musculoskeletal exposures and/or physiological responses as no standardized measures of acceptable quality were used or no clear information on reliability and/or validity was provided.

**Table 1:** Methodological quality assessment results.

Author	Study purpose	Study design	Study population	Musculoskeletal exposures and/or physiological responses	Musculoskeletal symptoms	Statistical analyses	Results	No. of positive categories	% of positive categories
Ahn et al (2016) [36]	+	+	?	-	?	-	+	3/7	42.9%
Albin and Mcloone (2014) [37]	-	+	?	?	?	?	-	1/7	14.3%
Billinghurst and Vu (2015) [38]	+	+	+	?	NA	?	?	3/6	50.0%
Chiang and Liu (2016) [33]	+	?	+	?	?	?	?	2/7	28.6%
Chiu et al (2015) [35]	+	+	+	?	+	+	+	6/7	85.7%
Choi et al (2016) [39]	?	+	+	-	NA	+	-	3/6	50.0%
Guan et al (2015) [40]	-	+	+	?	NA	?	+	3/6	50.0%
Guan et al (2016) [34]	+	+	?	?	NA	+	?	3/6	50.0%
Hong et al (2013) [22]	?	-	+	?	NA	?	?	1/6	16.7%
Inal et al (2015) [41]	+	+	+	?	?	?	+	4/7	57.1%
Jacquier-Bret et al (2014) [42]	?	?	-	?	NA	+	+	2/6	33.3%
Jung et al (2016) [43]	-	+	?	?	NA	?	+	2/6	33.3%
Kee et al (2016) [44]	+	+	+	?	?	+	+	5/7	71.4%
Kietrys et al (2015) [20]	+	+	+	?	NA	+	+	5/6	83.3%
Kim (2015) [45]	+	-	+	?	?	+	+	4/7	57.1%
Kim and Kim (2015) [25]	-	+	?	-	-	-	+	2/7	28.6%
Kim et al (2012) [46]	-	?	+	?	NA	-	-	1/6	16.7%
Kim et al (2014) [18]	+	+	+	?	?	+	+	5/7	71.4%
Kim et al (2014) [47]	+	+	+	?	?	+	+	5/7	71.4%
Kingston et al (2016) [48]	+	+	+	?	NA	+	+	5/6	83.3%
Ko et al (2016) [49]	+	+	+	?	NA	+	+	5/6	83.3%
Lee and Seo (2014) [50]	+	+	?	?	NA	+	+	4/6	66.7%
Lee et al (2012) [51]	-	+	+	?	?	?	-	2/7	28.6%
Lee et al (2015) [52]	+	+	+	?	NA	+	-	4/6	66.7%
Liang and Hwang (2016) [53]	+	?	+	?	NA	+	+	4/6	66.7%
Lin et al (2015) [54]	+	+	+	?	?	+	+	5/7	71.4%
Ning et al (2015) [55]	+	+	?	?	NA	?	+	3/6	50.0%
Park et al (2015) [56]	+	+	?	-	NA	+	?	3/6	50.0%
Pereira et al (2013) [57]	+	+	+	?	NA	+	+	5/6	83.3%
Shan et al (2013) [24]	+	+	+	?	?	+	+	5/7	71.4%
Shim (2012) [58]	?	?	+	?	NA	+	+	3/6	50.0%
Shin and Kim (2014) [59]	-	?	+	?	?	-	-	1/7	14.3%
Sommerich et al (2007) [23]	?	+	?	?	?	?	?	1/7	14.3%
Stoffregen et al (2014) [60]	?	+	+	?	NA	+	-	3/6	50.0%
Straker et al (2008) [19]	+	+	+	?	NA	+	+	5/6	83.3%
Trudeau et al (2012) [61]	+	+	?	?	NA	+	+	4/6	66.7%
Trudeau et al (2013) [62]	+	+	+	?	?	+	+	5/7	71.4%
Trudeau et al (2016) [63]	+	+	+	?	NA	+	+	5/6	83.3%
Vasavada et al (2015) [64]	+	+	+	?	NA	+	+	5/6	83.3%
Werth and Babski-Reeves (2014) [16]	?	+	+	?	NA	?	-	2/6	33.3%
Xie et al (2016) [21]	-	+	+	?	?	+	+	4/7	57.1%
Xiong and Muraki (2014) [65]	+	+	+	?	NA	?	?	3/6	50.0%
Xiong and Muraki (2016) [66]	+	+	?	?	NA	+	+	4/6	66.7%
Young et al (2012) [67]	+	+	+	?	NA	+	+	5/6	83.3%
Young et al (2013) [68]	+	?	+	?	NA	+	+	4/6	66.7%

(+): positive, (-): negative, (?): unclear (i.e. insufficient information available), NA: not applicable

### 3.4 Main findings

Musculoskeletal symptoms associated with MTSD use were reported in 17 studies (see S3 File, S4 File and S5 File), and included self-reported pain, discomfort or perceived comfort at the neck/shoulder, back and upper extremities (e.g. upper arm, forearm, wrist, fingers and thumb). Symptoms were measured either using a visual analogue scale, numeric or 100-point rating scale, Likert scale, Borg’s category ratio scale, body map or questions on symptom presence. Hypesthesia or dysesthesia in areas controlled by the median nerve, and temporomandibular disorders were examined via clinical tests in two of the studies [44, 51].

Musculoskeletal exposures (postures and muscle activity) associated with MTSD use were assessed in 38 studies (see S6 File, S7 File and S8 File). Posture variables examined included angles of head, neck, cranio-cervical, shoulder, distal upper extremity (e.g. elbow, wrist, fingers and thumb) flexion/extension, head and neck gravitational demand as well as posture and movement variability. These variables were measured using motion analysis systems, video or photograph analyses, range of motion meters or electrogoniometers. Muscle activity variables included electromyography (EMG) measurements of upper trapezius, cervical extensors and distal upper extremity muscles (e.g. wrist, finger or thumb flexors/extensors).

Physiological responses associated with MTSD use were examined in 11 studies and included measurements of pressure pain threshold, muscle fatigue, perceived exertion, cervical repositioning error, pinch strength, hand function and median nerve size (see S9 File and S10 File).

#### 3.4.1 Musculoskeletal symptoms associated with MTSD use

Musculoskeletal symptoms have been examined in four cross-sectional [23–25, 33], four case-control [21, 41, 44, 51] and eight experimental laboratory studies [18, 35–37, 47, 54, 59, 62]. In the cross-sectional studies, neck and/or shoulder symptoms had the highest prevalence rates reported among MTSD users, ranging from 26.3% to 60% [23, 25, 33]. One study had 37.5% and 30% of college students reporting symptoms related to tablet use at neck and shoulder respectively [33]. In another study, in a group of high school students, 50% or more of the students reported discomfort in the neck, and upper and lower back with tablet use [23]. In another large survey among high school students in China (n = 3,016), neck/shoulder pain was significantly associated with tablet use (OR 1.311, 95% CI 1.117–1.538), but not with the amount of daily tablet usage after controlling for confounding factors [24]. Another study (n = 80) did not find any statistically significant relationship of discomfort with tablet daily usage [33]. In another survey, higher (but not significant) prevalence of pain in various body regions was reported for participants who used a smartphone for more than two hours daily compared to those who used for less than two hours daily [25].

Three cross-sectional studies [23, 25, 33] have also reported associations of musculoskeletal symptoms with various aspects of MTSD use, i.e. gross postures, screen size and gaming task. One study reported higher prevalence of pain in those who used a smartphone whilst sitting and lying on their back compared to those who used a smartphone in other postures [25]. In another study, the frequency of awkward postures when using a tablet was significantly correlated with discomfort [23]. Smartphone screen size was also significantly but weakly correlated with pain in the waist. Another study found that participants who tended to play games on a tablet reported a higher prevalence of discomfort after using a tablet [33].

The case-control laboratory study by Xie and colleagues [21] showed that participants with and without neck symptoms developed discomfort after using a smartphone for ten minutes while sitting; however, the symptomatic group developed significantly more discomfort than the asymptomatic group.

Symptoms of the neck and/or shoulder were also shown to be associated with MTSD use in experimental laboratory studies. Pain in the neck increased over time after using a smartphone on a desk and on the lap [59]. Discomfort of the neck and shoulder were found to be significantly associated with different workstations; with the highest discomfort during tablet use on the lap, followed by during inclined sitting on a bed (with the tablet on lap) and on a desk [54]. Tablet tilt angles also had a significant effect on comfort of the neck; with decreasing comfort with decreasing (more horizontal) tilt angles [35]. During gaming, there was lower perceived comfort of the shoulder but not the neck when compared to watching movies [35].

Discomfort or pain in the distal upper extremities were also reported to be associated with MTSD use, including with workstations, features (e.g. tilt angles, key size) and tasks in one case-control and several experimental studies. In a case-control study conducted with university students (n = 102), self-reported pain of the hand was significantly higher in the high smartphone “addiction” group compared to the low “addiction” group [41]. Experimental laboratory studies showed significantly lower comfort at distal upper extremities when typing on a virtual compared to a physical keyboard on a laptop [18]. Discomfort of the wrists and arms were generally higher with tablet use on the lap or during inclined sitting on a bed compared to when sitting at a desk [54]. Comfort of the hands and arms was reported to be lower at 60° compared to at 34° tablet tilt angles [37]. There was also lower distal upper extremity comfort with small compared to large key sizes [47], and during gaming compared to movie watching [35].

Upper and lower back, and buttock discomfort were also reported in experimental laboratory studies, with one study showing higher discomfort when using a tablet on the lap compared to when sitting inclined on a bed or at a desk; while no significant differences in discomfort among different keyboard designs were shown [54]. Higher overall discomfort (without specifying the body region) during tablet use in landscape compared to portrait orientation, and standard compared to split keyboard design (tablet in landscape orientation) were found in the study by Trudeau and colleagues [62].

Other musculoskeletal symptoms were found to be associated with smartphone use in two other case-control laboratory studies [44, 51]. There was more frequent presentation of temporomandibular problems [44] and increased wrist/forearm sensitivity [51] in smartphone “addicted” compared to “non-addicted” participants.

#### 3.4.2 Musculoskeletal exposures associated with usage of MTSD

Musculoskeletal exposures have been reported for the amount of MTSD use and in comparisons with other device use in one cross-sectional and several laboratory studies. In a cross-sectional study among university students (n = 426), of which more than 90% used a mobile phone (97.7% of which were a smartphone) for more than one hour per day [34], no significant association between head and neck postures (both during usual standing posture and while looking at a smartphone in standing) and frequency of mobile phone usage were found.

Two case-control laboratory studies revealed associations of higher smartphone usage and smartphone “addiction” with greater neck flexion [43, 56] and rounded shoulders during habitual standing [43]. In addition, two other case-control laboratory studies found greater upper and lower cervical flexion [45], and greater muscle activity in cervical erector spinae and upper trapezius muscles in a group with neck/shoulder symptoms compared to an asymptomatic group [21].

The experimental laboratory studies generally showed higher head and neck flexion and muscle activity during MTSD use. In two experimental laboratory studies by Guan and colleagues [34, 40] with a large number of participants (n = 186 and 429), significantly greater extents of static flexed head and neck and forward head posture were found during standing while looking at a smartphone (handholding smartphone) compared to normal standing (without holding smartphone); head and neck flexion were also greater in males compared to females during both standing while looking at a smartphone and normal standing.

In other experimental laboratory studies, MTSD use was compared with the use of traditional electronic devices. In a study by Straker and colleagues [19] among young children, there were significantly higher head and neck flexion, higher shoulder flexion and elevation, and lower cranio-cervical and cervico-thoracic angles when using a tablet with a stylus compared to when using a desktop computer. The use of a tablet also induced greater variability of neck posture and muscle activity than when using a desktop computer. Higher neck flexion when using a tablet compared to a laptop or netbook has also been reported, but not when compared to a physical keypad phone [20].

Muscle activity of the upper trapezius and/or cervical erector spinae were also found to be higher while using a tablet compared to a desktop computer [19], or when using a virtual compared to a physical keyboard on a laptop [18]. However, trapezius muscle activity was lower during the use of a smartphone when compared to a desktop computer [21], while no significant differences were found when smartphone use was compared to physical keypad phone use [20].

Postures and muscle activity of the distal upper extremities were also dependent on the types of devices, with generally greater non-neutral postures, but lower muscle activity at the level of the wrist, elbow, fingers and/or thumb found during use of MTSD compared to traditional electronic devices. There was more wrist extension [16, 20], wrist pronation and elbow flexion [48] during the use of a smartphone or tablet compared to a laptop or physical keypad phone. Several studies reported similar findings of lower muscle activities at wrist and finger flexors and extensors [16, 20–22] and thumb abductors [20, 22] when using a smartphone or tablet compared to a laptop, netbook or physical keypad phone, and when using a virtual compared to physical keyboard [18].

#### 3.4.3 Musculoskeletal exposures associated with features of MTSD

Musculoskeletal exposures associated with different features of MTSD (e.g. screen size or tilt angles) were examined in several experimental and case-control laboratory studies. Greater non-neutral postures and higher muscle activity were generally found with larger tablet screen size. A significant trend for increasing head/neck flexion with increasing screen size (3.5 inch smartphone, 7 and 9.5 inch tablet) [20], and generally greater amount of wrist extension and ulnar deviation as well as muscle activity of upper trapezius, wrist and finger flexors and extensors during the use of a larger compared to a smaller tablet screen size [20, 57] were reported. Additionally, it was noted that placing MTSD on one’s lap was most adopted when using a device with a larger screen (9.5 inch tablet), while handholding and texting with both thumbs was most preferred when using a device with a smaller screen (3.5 inch smartphone) [20].

Tilt angles of tablet supported in cases on a desk also had an effect on musculoskeletal exposures associated with MTSD use, and were investigated in some relatively small studies (n = 10 to n = 33) conducted with young to middle-aged adults. Four studies showed, in general, increasing head and neck flexion with decreasing tablet tilt angles ranging from 73° tilt (almost vertical) to 0° tilt (horizontal on desk) [33, 37, 64, 67]. The gravitational demand on head and neck, trunk flexion angles [64] and activity of the upper trapezius were higher, but anterior deltoid muscle activity was lower [35], while using a tablet at lower compared to higher tilt angles. No differences were found for finger flexors muscle activity [35] and hand gestures [38] among different tilt angles. Other features of MTSD use, e.g. key sizes, keyboard design and location, were also found to affect musculoskeletal exposures [47, 54, 62].

#### 3.4.4 Musculoskeletal exposures associated with tasks on MTSD

Various tasks that could be performed on a MTSD (e.g. typing, gaming, internet browsing, reading, watching videos) have been investigated in some experimental laboratory studies, with task duration ranging from 90 seconds to 15 minutes. No case-control or cross-sectional studies examining the effect of different tasks on a MTSD were found. The tasks on MTSDs can be considered as active tasks, when frequent finger activity is required (e.g. typing, gaming), or passive tasks, which consist mainly of visual interaction with minimal or no finger activity (e.g. internet browsing, reading, watching videos). Active tasks were generally associated with greater non-neutral postures and muscle activity of the head, neck and distal upper extremities compared to passive tasks. For example, typing or gaming was associated with significantly higher head and neck flexion [33, 52, 55], and higher muscle activity of upper trapezius, anterior deltoid [35, 68] and cervical extensors [55] compared to passive tasks. Another study however did not find any significant differences in postures and gravitational demand at the head and neck between typing and reading tasks [64]. The authors of the latter study did not standardize duration for each of the tasks, which may have affected the accuracy of findings. At the distal upper extremities, greater wrist pronation and ulnar deviation [48, 68], as well as greater muscle activity of finger flexors [35, 55] and wrist extensors and flexors [68], were found during active tasks compared to passive tasks.

#### 3.4.5 Musculoskeletal exposures associated with positions of MTSD use

Different positions of MTSD use, affected by the gross body postures (such as sitting, standing), handhold positions (such as MTSD handheld at different levels, one/two-handed hold or handheld in portrait/landscape orientation) and workstations (such as MTSD on desk, lap or sofa), were examined in one cross-sectional study [53], one case-control [21] and several experimental laboratory studies. The cross-sectional study observed handhold positions and general postures of passengers using smartphones while commuting in metro trains in Taiwan [53]. They found that the use of two hands, with one hand holding and the other hand operating the smartphone, was most commonly observed in sitting postures (45.5%). Operating the smartphone with only one hand, with the other hand holding a train pole or hand strap, was most commonly observed in standing commuters (45.8%).

Two experimental laboratory studies compared exposures during smartphone use in different gross body postures. One study reported significantly higher head flexion while using a smartphone in sitting compared to use in standing [52]. The other study found no significant differences in muscle activity of splenius capitis and upper trapezius, among sitting postures with neutral, “middle” or “maximum neck bending” with five minutes of smartphone use [39].

Different MTSD handhold positions were examined in one case-control and some experimental laboratory studies. One experimental laboratory study found significantly higher neck and elbow flexion while handholding a smartphone at chest level, compared to at eye or knee level (with the trunk bent forward and elbows resting on the thighs) [49]. Muscle activity of the upper trapezius muscle was, however, significantly higher at eye level than at knee and chest level. There was a small but significantly lower head/neck flexion of 2° during one-handed compared to two-handed texting on a smartphone [20]. Upper trapezius muscle activity was however, significantly higher during one-handed compared to two-handed hold of a tablet [68], with comparable (but non-significant) findings in another study [21]. Similarly, muscle activity was higher for wrist extensors [20–22, 68], wrist flexors [20, 22], fingers and thumb muscles [21, 22, 49] during one-handed compared to two-handed hold. The different handholds caused no significant differences in distal upper extremity postures [20, 68]. Postures at wrist and thumb during the different handholds were influenced instead by MTSD orientation, with higher wrist extension and thumb movements during two-handed hold in landscape than one-handed hold in portrait orientation [63]. During the use of a tablet in portrait compared to landscape orientation, there were significantly lower wrist, fingers and thumb joint postural angles [57, 62].

Different workstations for MTSD use, i.e. a desk or use of non-traditional workstations (such as bed, sofa or lap), were compared in some experimental and case-control laboratory studies. It was generally found that neck flexion was significantly greater during tablet use in non-traditional workstations (i.e., sofa [16] or lap [64, 67]) compared to when sitting at a desk. Gravitational demand on the head and neck were also significantly higher while using a tablet on the lap compared to on a desk [64]. Muscle activity of the upper trapezius, however, was lower during tablet use on the lap compared to on a desk, which corresponded to lower shoulder flexion and elevation [68]. Another study showed no differences in flexion relaxation ratio of cervical erector spinae muscle activity between smartphone use on a desk and the lap [59]. For distal upper extremities postures, there was generally higher elbow flexion [16, 54], wrist extension [54, 68] and ulnar/radial deviation acceleration [68] but no differences in wrist muscle activity [68] while using a tablet on non-traditional workstations (sofa, lap or bed) compared to on a desk.

#### 3.4.6 Physiological responses associated with MTSD use

Physiological responses (e.g. pressure pain threshold, muscle fatigue, perceived exertion, cervical repositioning error, pinch strength, hand function, median nerve size), were investigated in experimental and case-control laboratory studies, and were found to be associated with MTSD use. These studies generally showed increased physiological responses with MTSD use, suggesting an increased risk for musculoskeletal symptoms. Pressure pain threshold in the upper trapezius muscle was significantly lower in a group with heavy smartphone “addiction” compared to a control group with low “addiction” [56], and after using a smartphone compared to before [46]. Muscle fatigue (measured by EMG) of upper trapezius, elbow and wrist flexors, and thumb abductors were higher after using a smartphone compared to before [46], while that of upper trapezius and splenius capitis muscles also increased during smartphone use in sitting with “maximum” compared to “middle neck bending” [39]. Self-reported fatigue in neck, shoulder, forearm and wrist were also affected by features of a tablet, i.e. screen size, grip and stylus shape [57].

Perceived exertion was higher in symptomatic participants (with neck/shoulder discomfort) compared to those who were asymptomatic after using a smartphone [21]. In other case-control studies, high smartphone “addiction” or usage was found to be associated with greater cervical repositioning errors [50], reduced pinch strength and hand function [41]. In an experimental laboratory study, median nerve circumference and area (examined via ultrasonography) decreased significantly after using a smartphone compared to before [58]. Two case-control laboratory studies showed significantly higher median nerve cross-sectional area in the dominant arm side compared to non-dominant side in the high smartphone “addiction” group [41], while no significant differences in median nerve thickness among groups with different degrees of smartphone “addiction” were found [51].

## 4 Discussion

### 4.1 Discussion of main findings

This is the first review, to our knowledge, to systematically describe the available evidence on musculoskeletal symptoms and musculoskeletal exposures associated with MTSD use. Findings from this review, based on evidence mainly from experimental and case-control laboratory studies with only a few cross-sectional studies, suggest that aspects of MTSD use (i.e. usage, features, tasks and positions) can create various musculoskeletal exposures, possibly leading to physiological responses and musculoskeletal symptoms. However, current evidence is limited with many studies of low methodological quality with limitations in measurement methods, study design and/or presentation of the results. Moreover, a wide variation in measures and conditions of MTSD use evaluated across the studies makes it hard to make valid comparisons and to draw firm conclusions on certain aspects of MTSD use. No longitudinal studies were identified, and thus the direction of the associations as well as the long-term musculoskeletal impact of MTSD use were also not able to be determined.

#### 4.1.1 Musculoskeletal symptoms associated with MTSD use

Current evidence on musculoskeletal symptoms associated with MTSD use from cross-sectional studies is limited with mainly low quality studies. However, available evidence does suggest that MTSD use and aspects of its use, i.e. long duration, awkward postures, larger screen size and gaming task, may be associated with musculoskeletal symptoms [24, 25, 33], most commonly in the neck/shoulder region [23–25]. Limitations of the available studies include the use of measures of the amount and aspects of MTSD use, and musculoskeletal symptoms, with no reliability and/or validity of the measurement methods specified by the authors. Confounding factors were often not considered, and representative samples were not obtained in many of the studies. These limitations may have affected the accuracy of findings in the studies and potentially biased the true associations between musculoskeletal symptoms and MTSD use.

From case-control and experimental laboratory studies, there was consistent evidence that musculoskeletal symptoms are associated with MTSD use, and different positions, features and tasks of its use can have an impact on the symptoms experienced [35, 37, 47, 54]. Other musculoskeletal symptoms in areas controlled by the median nerve may also be associated with greater usage of MTSD or smartphone “addiction” [51], suggesting risk for ‘repetitive strain injuries’ or ‘carpal tunnel syndrome’ reported with traditional desktop computer use [69, 70] may also be a risk with MTSD use. Nonetheless, such evidence is still limited with only a small number of studies examining symptoms associated with MTSD use, and measures with unspecified reliability and validity, which may have biased the findings.

#### 4.1.2 Musculoskeletal exposures associated with usage of MTSD

Evidence from the few experimental laboratory studies showed that musculoskeletal exposures of greater non-neutral postures [34, 40] and gravitational demand [64] at the head and neck/shoulder were associated with MTSD use when compared to not using any device. When compared to the more traditional electronic devices (e.g. physical keypad phone, desktop/laptop computers), exposures at head and neck during MTSD use may differ which may be due to the different device placement and the use of a touch screen interface. For example, greater non-neutral postures and muscle activity at the head and neck were found with tablet (placed flat on a desk) compared to desktop computer use (supported upright on a desk) [19], while no significant differences were found between smartphone and physical keypad phone, which were both used handheld [20]. The different placements and viewing angles with tablet and desktop computer use is likely to have affected the amount of neck bending. Moreover, the use of a touch screen has been shown to be associated with increased upper trapezius muscle activity, which may be due to wrists and fingers not being able to fully rest on the screen [17, 18]. Therefore, current evidence indicates that different device placement and the use of a touch screen interface can influence musculoskeletal exposures, and cause greater non-neutral postures at head/neck [19] and distal upper extremities during MTSD use [16, 20–22], which may lead to increased risk of symptoms.

#### 4.1.3 Musculoskeletal exposures associated with features, tasks and positions of MTSD

Current evidence showed that screen size had an effect on musculoskeletal exposures, potentially through an influence on weight and placement position of the device. MTSD with a larger screen may pose greater stress at the neck/shoulder and distal upper extremities [20, 57], possibly due to heavier weight of the larger screen, and a preference to place it on the lap (which tended to induce greater head/neck flexion and muscle activity). Handholding a larger screen could also create greater shoulder and arm fatigue [20]. Hence, it may be more advisable to support a larger screen MTSD on a desk rather than handholding it, especially for prolonged periods of use.

There was also consistent evidence from experimental laboratory studies that lower tablet tilt angle (with tablet supported flat on a surface) generally cause greater gravitational demand [64], muscle activity [35] and non-neutral postures at the head/neck [33, 37, 64, 67] than higher tablet tilt angles, with the greatest exposures at 0° tilt (placed flat on surface). At lower tilt angles, the head and neck need to bend forward more to look at the screen which poses more biomechanical stresses at the head and neck/shoulder. However, exposures on upper extremities during different MTSD tasks should also be considered when choosing an optimal MTSD tilt angle [33]. Although there is less non-neutral head and neck/shoulder postures at higher tilt angles of e.g. 60° or 70° (which might be appropriate during passive tasks), such high tilt angles may not be conducive for tasks that require frequent finger input (e.g. typing) and may induce greater non-neutral postures and muscle activity at shoulder, wrist and/or fingers. Tilt angles ranging from 33° to 37° were preferred during various tasks on a tablet [33, 37, 67], which may be the angle range that is more comfortable for wrists and fingers during active tasks. Therefore, higher tablet tilt angles (with tablet supported on a surface) may tend to result in lesser biomechanical stresses at the head and neck than lower tilt angles but may not be as ideal for exposures at distal upper extremities, especially during active tasks.

Current evidence also showed that active MTSD tasks generally caused greater non-neutral postures and higher muscle activity around the head, neck, wrist and finger areas, which pose more risk for biomechanical stresses compared to passive tasks [33, 35, 52, 68]. However, active tasks may provide movement variation. It has been suggested that variation may be a strategy to reduce the risk for musculoskeletal symptoms from increased exposures [71, 72]. However, it is still unknown if active MTSD tasks can provide sufficient movement variation to offset the increased risk for symptoms from non-neutral postures and higher muscle activities.

Different handhold positions during MTSD use also had an effect on musculoskeletal exposures. One-handed hold tended to pose more strain with higher muscle activity on wrists, fingers and thumbs compared to two-handed hold [20–22, 49, 68]. However, handhold positions also varied with device placement, so there is a need to consider this interaction and balance of exposures at the head/neck versus upper extremities in reducing musculoskeletal strain during use of MTSDs.

Evidence on workstations of MTSD use suggests that use in a sitting position in non-traditional workstations (i.e. on a sofa, bed or lap) may pose more musculoskeletal stress on the head and neck compared to supported use on a desk [16, 54, 64, 67]. Lower muscle activity at upper trapezius was however reported during the use of a tablet placed on the lap compared to on a desk, possibly due to the more pronounced arm and shoulder postures at the desk versus on the lap [68]. Nonetheless, these studies have only looked at a limited number of sitting positions in non-traditional workstations (i.e., bed, sofa or lap) whereas other workstations (e.g. on the floor) and a variety of postures at those workstations (e.g. side lying, prone lying, cross-legged sitting) [16, 73], along with the resulting musculoskeletal consequences, are yet to be studied.

Overall, current evidence from case-control and experimental laboratory studies suggests that features, tasks and positions of MTSD use significantly affect musculoskeletal exposures associated with MTSD use, and can interact with each other to influence the exposures. However, some studies had more than one independent variable in each condition of MTSD use, which makes comparisons among studies and drawing strong conclusions difficult.

#### 4.1.4 Physiological responses associated with MTSD use

Evidence from case-control and experimental laboratory studies also suggests that MTSD use (and aspects of MTSD use such as different MTSD features, the amount of usage and extent of MTSD “addiction”) can cause physiological responses (such as median nerve changes, lower pressure pain threshold or greater muscle fatigue) which suggest potentially increased susceptibility to musculoskeletal symptoms [41, 56–58]. For example, the median nerve changes reported with MTSD use [41, 58] suggest that MTSD use may have the potential to lead to narrowing of [74, 75], and increased pressure in the carpal tunnel [76], and thus pose a risk for carpal tunnel syndrome [77].

#### 4.1.5 Association between musculoskeletal symptoms and exposures in MTSD use

As summarised above, there is some evidence from cross-sectional, case-control and experimental laboratory studies that musculoskeletal symptoms, musculoskeletal exposures and physiological responses are each related to MTSD use. The findings on musculoskeletal exposures and physiological responses associated with MTSD use are also consistent with current understanding of increased risk and susceptibility for musculoskeletal symptoms. Indeed, some of the included studies suggested increased non-neutral postures, and/or increased muscle activity were potential mechanisms for the increased discomfort or pain reported from MTSD use [21, 35, 37, 44, 54, 62]. Nonetheless, evidence on what exposure aspects are the critical mechanisms for the observed associations between musculoskeletal symptoms and MTSD use is still limited.

#### 4.1.6 Influence of individual differences on musculoskeletal symptoms and exposures

Individual differences in musculoskeletal symptoms and exposures associated with MTSD use between symptomatic and asymptomatic participants, between participants with different hand sizes and shapes, and between males and females were also examined in a few of the studies. Musculoskeletal symptoms and exposures (cervical flexion and neck/shoulder muscle activity) were greater in participants with neck/shoulder symptoms compared to those who were asymptomatic [21, 45]. MTSD use may hence pose even greater risk for biomechanical stress and load on individuals who are symptomatic, inducing a vicious cycle of musculoskeletal symptoms. This has been reported before in studies showing altered motor control and muscle activation (during non-MTSD tasks) among people with neck pain [78–80]. Different hand sizes and shapes may also influence musculoskeletal symptoms or exposures at the distal upper extremity, as one study indicated their significant effect on muscle activity of the fingers and thumb during MTSD use [36].

Gender differences were also noted with higher head and neck flexion found in males compared to females during smartphone use [34, 40], as previously noted during computer use [81]. This may be due to taller statures of males than females [82]. However, the risk of neck/shoulder [24, 83] and low back pain [24] have been shown to be higher instead in females compared to males, possibly due to higher pain sensitivity in females [84] or their different nature of computer use (e.g. type of activities, duration) [1, 83]. These findings suggest different musculoskeletal risks involved with MTSD use between males and females, and a potential modifying effect of gender on associations between symptoms and MTSD use.

### 4.2 Quality of evidence

The quality of the included studies was generally low, with less than one third of the studies scoring 50% or above on the methodological quality assessment, mainly due to insufficient or unclear information provided on measurement methods of musculoskeletal symptoms and/or exposures in many of the studies. Although some methods for the assessment of musculoskeletal exposures were sometimes thoroughly described (e.g. EMG or motion analysis), reliability and validity of the measures were often not specified. In addition, anatomical points for measuring postures and muscle activity were not always clearly indicated and various terminologies for postural angles were used, making it hard to consolidate findings from the studies. Some of the studies were also unclear in reporting of the results. Moreover, as a number of studies did not report on symptoms for each specific body region (by only providing an average or overall score), it was difficult to determine the impact of MTSD use on specific body regions.

### 4.3 Further research

Findings from the current review need to be interpreted with caution as current evidence is limited and mainly from cross-sectional, case-control and experimental laboratory study designs with poor methodological quality. High quality epidemiological studies, including longitudinal studies, examining MTSD use in natural settings (e.g. in the home or school environment) are therefore needed to strengthen the body of evidence. To date however, no low burden but accurate methods for measuring the amount and nature of MTSD usage in daily life are available and there is a need to develop reliable and valid methods to capture MTSD usage accurately. Laboratory studies should use accurate measurement methods for musculoskeletal symptoms and exposures (with established validity and reliability), and anatomical angle definitions should be clearly specified and preferably be harmonized across studies. MTSD use in other possible positions and non-traditional workstations and prolonged duration of MTSD use should also be investigated. It will also be important to examine variability of musculoskeletal exposures during MTSD use and gender differences.

This review is the first, to our knowledge, to systematically describe the current evidence on musculoskeletal symptoms and exposures associated with MTSD use. [85]., our review was limited. Narrative synthesis rather than meta-analysis was used in the review due to the heterogeneity of study designs, methods, outcomes and data presented. The systematic approach used to screen articles, extract data and assess methodological quality of the included studies helped to minimise biases which may be introduced in unstructured narrative reviews where findings from one study may be given inappropriate emphasis. A further limitation was that the methodological quality assessment tool used may not have captured all the possible issues that may be present across the broad range of study designs in the included studies; nonetheless, the tool used was the most appropriate available for the purposes and nature of studies in this review.

This review has excluded studies that examined gait and balance parameters during MTSD use. It will be important to review this evidence, considering the portability of MTSDs and their use during walking and multitasking [86, 87]. Findings from the included studies on performance, task efficiency or typing speed during MTSD use were also not reported in this review. These outcomes should be considered as they provide useful information on productivity associated with MTSD use.

### 4.4 Implications for wise use of MTSDs

Based on currently limited available evidence on MTSD use, and other research on risk factors for musculoskeletal symptoms, some tentative suggestions for wise use of MTSD to help reduce musculoskeletal exposures and associated risks for musculoskeletal symptoms from MTSD use are proposed (Table 2).

**Table 2:** Tentative suggestions for wise use of MTSD.

▪ Avoid excessive total usage ▪ Avoid prolonged static postures ▪ Use opportunities to vary whole body, head/neck and upper extremity postures during MTSD use ▪ Avoid awkward postures during prolonged or repetitive use ▪ Position MTSD at a height to balance head/neck and upper extremity stress–holding a MTSD at around eye level encourages neutral head/neck posture but increases upper extremity loading; holding a MTSD at around waist/lap level increases head/neck flexion but reduces upper extremity loading ▪ For longer durations of use, support MTSD at a tilt angle (e.g. with the use of device accessories) to balance head/neck and upper extremity stress–a higher tilt encourages neutral head/neck posture and is good for viewing only tasks; a lower tilt allows lower wrist and finger stresses and is good for tasks requiring finger or thumb input ▪ Avoid high repetition of movements such as prolonged typing or swiping on MTSD ▪ Avoid forceful exertions such as holding larger or heavy MTSD in one hand for long durations

## 5 Conclusion

There is limited evidence that MTSD use, and various aspects of its use (i.e. amount of usage, features, tasks and positions), are associated with musculoskeletal symptoms and exposures. This is due to mainly low quality experimental and case-control laboratory studies, with few cross-sectional and no longitudinal studies. Further research with higher quality studies which examine: MTSD dose-response relationship, associations of specific aspects of MTSD use (e.g. features, tasks and positions) with musculoskeletal symptoms, as well as the mechanisms, direction and long-term effects of the associations are required. This enhanced evidence is needed to provide evidence-based guidelines for wise use of MTSDs.

## Supporting information

S1 File Search strategy. (DOCX)

S2 File Methodological quality assessment list. (DOCX)

S3 File Summary of included *cross-sectional* studies (MTSD use and *musculoskeletal symptoms*). (DOCX)

S4 File Summary of included *case-control laboratory* studies (MTSD use and *musculoskeletal symptoms*). (DOCX)

S5 File Summary of included *experimental laboratory* studies (MTSD use and *musculoskeletal symptoms*). (DOCX)

S6 File Summary of included *cross-sectional* studies (MTSD use and *musculoskeletal exposures*). (DOCX)

S7 File Summary of included *case-control laboratory* studies (MTSD use and *musculoskeletal exposures*). (DOCX)

S8 File Summary of included *experimental laboratory* studies (MTSD use and *musculoskeletal exposures*). (DOCX)

S9 File Summary of included *case-control laboratory* studies (MTSD use and *physiological responses*). (DOCX)

S10 File Summary of included *experimental laboratory* studies (MTSD use and *physiological responses*). (DOCX)

S11 File PRISMA checklist. (DOC)
