# Supplementary material for: The associations of mobile touch screen device use with musculoskeletal symptoms and exposures: A systematic review
Source: PLoS One. 2017 Aug 7;12(8):e0181220. doi: 10.1371/journal.pone.0181220 (PMC5546699; doi:10.1371/journal.pone.0181220)
Supplement: S1 File — (DOCX) [file pone.0181220.s001.docx]

**S1. Search strategy**

| **Database** | **EMBASE** | |
| --- | --- | --- |
| **Date** | **13/6/2016** | |
| **Strategy** | **#1 AND #2 AND #3** | |
| **#1** | Mobile touch screen devices | Minicomputer/ OR Microcomputer/ OR Mobile phone/ OR Information technology/ OR ("touch screen*" OR "touchscreen*" OR "touch-screen*" OR "touch interface*" OR "touch technolog*" OR "tablet comput*" OR "slate comput*" OR "mobile comput*" OR "portable comput*" OR “tablet PC*” OR  "smart phone*" OR "smartphone*" OR "smart-phone" OR “mobile phone*” OR “cell* phone*” OR "mobile technolog*" OR "mobile device*" OR "information communication* technolog*" OR "information technolog*" OR "information and communication* technolog*" OR "screen time" OR "screen us*" OR "screen based activit*" OR "screen-based activit*" OR "screen activit*").ti,ab,kw. |
| **#2** | Posture muscle activity musculoskeletal symptoms | Body posture/ OR Skeletal muscle/ OR Musculoskeletal system/ OR Musculoskeletal injury/ OR Muscle injury/ OR Electromyography/ OR Motor activity/ OR "movement (physiology)"/ OR Body movement/ OR Musculoskeletal function/ OR Musculoskeletal pain/ OR Pain/ OR Back ache/ OR Neck pain/ OR  Low back pain/ OR (“posture*” OR "electromyograph*" OR "motor activit*" OR "muscle activit*" OR "musculoskeletal activit*" OR “discomfort” OR “symptom*” OR “pain”).ti,ab,kw. |
| **#3** | Limit to year 1993 (inclusive) onwards | limit to yr="1993-Current" |

| **Database** | **Medline** | |
| --- | --- | --- |
| **Date** | **13/6/2016** | |
| **Strategy** | **#1 AND #2 AND #3** | |
| **#1** | Mobile touch screen devices | Minicomputer/ OR Computers, handheld/ OR Human engineering/ OR Cell phones/ OR ("touch screen*" OR "touchscreen*" OR "touch-screen*" OR "touch interface*" OR "touch technolog*" OR "tablet comput*" OR "slate comput*" OR "mobile comput*" OR "portable comput*" OR “tablet PC*” OR  "smart phone*" OR "smartphone*" OR "smart-phone" OR “mobile phone*” OR “cell* phone*” OR "mobile technolog*" OR "mobile device*" OR "information communication* technolog*" OR "information technolog*" OR "information and communication* technolog*" OR "screen time" OR "screen us*" OR "screen based activit*" OR "screen-based activit*" OR "screen activit*").ti,ab. |
| **#2** | Posture muscle activity musculoskeletal symptoms | Posture/ OR Muscle, skeletal/physiology OR Musculoskeletal system/ OR Movement/physiology OR Electromyography/ OR Motor activity/physiology OR Musculoskeletal pain/ OR Pain/ OR Back pain/ OR Low back pain/ OR Neck pain/ OR (“posture*” OR "electromyograph*" OR "motor activit*" OR "muscle activit*" OR "musculoskeletal activit*" OR “discomfort” OR “symptom*” OR “pain”).ti,ab. |
| **#3** | Limit to year 1993 (inclusive) onwards | limit to yr="1993 -Current” |

| **Database** | **PsycINFO** | |
| --- | --- | --- |
| **Date** | **13/6/2016** | |
| **Strategy** | **#1 AND #2 AND #3** | |
| **#1** | Mobile touch screen devices | Microcomputers/ OR Computer Peripheral Devices/ OR [Human Factors Engineering](http://ovidsp.tx.ovid.com.dbgw.lis.curtin.edu.au/sp-3.15.1b/ovidweb.cgi?&Controlled+Vocabulary=Mapping%7c1&Return=mapping&S=DHDNFPEBOCDDEKCNNCKKHCIBGOJIAA00)/ OR Cellular phones/ OR Mobile devices/ OR Computer usage/ OR Human computer interaction/ OR Information technology/ OR ("touch screen*" OR "touchscreen*" OR "touch-screen*" OR "touch interface*" OR "touch technolog*" OR "tablet comput*" OR "slate comput*" OR "mobile comput*" OR "portable comput*" OR “tablet PC*” OR "smart phone*" OR "smartphone*" OR "smart-phone" OR “mobile phone*” OR “cell* phone*” OR "mobile technolog*" OR "mobile device*" OR "information communication* technolog*" OR "information technolog*" OR "information and communication* technolog*" OR "screen time" OR "screen us*" OR "screen based activit*" OR "screen-based activit*" OR "screen activit*").ti,ab,id. |
| **#2** | Posture muscle activity musculoskeletal symptoms | Posture/ OR Musculoskeletal system/ OR Musculoskeletal disorders/ OR Electromyography/ OR Pain/ OR Back pain/ OR (“posture*” OR "electromyograph*" OR "motor activit*" OR "muscle activit*" OR "musculoskeletal activit*" OR “discomfort” OR “symptom*” OR “pain”).ti,ab,id. |
| **#3** | Limit to year 1993 (inclusive) onwards | limit #3 to yr=”1993 –Current” |

| **Database** | **Proquest** | |
| --- | --- | --- |
| **Date** | **14/6/2016** | |
| **Strategy** | **#1 AND #2 AND #3** | |
| **#1** | Mobile touch screen devices | TI,AB,IF( "touch screen*" OR "touch-screen*" OR touchscreen* OR "touch interface*" OR "touch technolog*" OR “tablet PC*” OR "tablet comput*" OR "slate comput*" OR "mobile comput*" OR "portable comput*" OR "cell* phone*" OR "mobile phone*" OR "hand phone*" OR "handphone*" OR "smartphone" OR "smart-phone" OR "smart phone" OR "mobile technolog*" OR "mobile device*" OR "screen time" OR "screen us*" OR "screen based activit*" OR "screen-based activit*" OR "screen activit*" OR "information technolog*" OR "information communication* technolog*" OR "information and communication* technolog*")   \|  \|  \| \| --- \| --- \| |
| **#2** | Posture muscle activity musculoskeletal symptoms | (TI,AB,IF("posture*" OR "electromyograph*" OR "motor activit*" OR "muscle activit*" OR "musculoskeletal activit*" OR "musculoskeletal disorder*" OR "discomfort" OR "symptom*" OR pain)) |
| **#3** | Limit to year 1993 (inclusive) onwards; exclude newspapers, trade journals, wire feeds, magazines, blogs podcasts and websites | (YR(>=1993) ((TI,AB,IF("touch screen*" OR "touch-screen*" OR touchscreen* OR "touch interface*" OR "touch technolog*" OR "tablet comput*" OR "tablet PC*" OR "slate comput*" OR "mobile comput*" OR "portable comput*" OR "cell* phone*" OR "mobile phone*" OR "hand phone*" OR "handphone*" OR "smartphone" OR "smart-phone" OR "smart phone" OR "mobile technolog*" OR "mobile device*" OR "screen time" OR "screen us*" OR "screen based activit*" OR "screen-based activit*" OR "screen activit*" OR "information technolog*" OR "information communication* technolog*" OR "information and communication* technolog*")) AND (TI,AB,IF("posture*" OR "electromyograph*" OR "motor activit*" OR "muscle activit*" OR "musculoskeletal activit*" OR "musculoskeletal disorder*" OR "discomfort" OR "symptom*" OR pain)))) NOT stype.exact("Newspapers" OR "Trade Journals" OR "Wire Feeds" OR "Magazines" OR "Blogs, Podcasts, & Websites") |

| **Database** | **Scopus** | |
| --- | --- | --- |
| **Date** | **14/6/2016** | |
| **Strategy** | **#1 AND #2 AND #3** | |
| **#1** | Mobile touch screen devices | TITLE-ABS-KEY ( "touch screen*"  OR  "touch-screen*"  OR  touchscreen*  OR  "touch interface*"  OR  "touch technolog*"  OR  "tablet comput*"  OR  "slate comput*"  OR  "mobile comput*"  OR  "portable comput*"  OR  "cell* phone*"  OR  "mobile phone*"  OR  "hand phone*"  OR  "handphone*"  OR  "smartphone"  OR  "smart-phone"  OR  "smart phone"  OR  "mobile technolog*" )  OR  TITLE-ABS-KEY ( "mobile device*"  OR  "screen time"  OR  "screen us*"  OR  "screen based activit*"  OR  "screen-based activit*"  OR  "screen activit*"  OR  "information technolog*"  OR  "information communication* technolog*"  OR  "information technolog*" )  OR  TITLE-ABS-KEY ( "information and communication* technolog*" ) |
| **#2** | Posture muscle activity musculoskeletal symptoms | TITLE-ABS-KEY ( "posture*"  OR  "electromyograph*"  OR  "motor activit*"  OR  "muscle activit*"  OR  "musculoskeletal activit*"  OR  "musculoskeletal disorder*"  OR  "discomfort"  OR  "symptom*"  OR  pain ) |
| **#3** | Limit to year 1993 (inclusive) onwards | ( TITLE-ABS-KEY ( "touch screen*"  OR  "touch-screen*"  OR  touchscreen*  OR  "touch interface*"  OR  "touch technolog*"  OR  “tablet PC*” OR "tablet comput*"  OR  "slate comput*"  OR  "mobile comput*"  OR  "portable comput*"  OR  "cell* phone*"  OR  "mobile phone*"  OR  "hand phone*"  OR  "handphone*"  OR  "smartphone"  OR  "smart-phone"  OR  "smart phone"  OR  "mobile technolog*" OR "mobile device*"  OR  "screen time"  OR  "screen us*"  OR  "screen based activit*"  OR  "screen-based activit*"  OR  "screen activit*"  OR  "information technolog*"  OR  "information communication* technolog*"  OR  "information technolog*" OR "information and communication* technolog*") ) AND ( TITLE-ABS-KEY ("posture*"  OR  "electromyograph*"  OR  "motor activit*"  OR  "muscle activit*"  OR  "musculoskeletal activit*"  OR  "musculoskeletal disorder*"  OR  "discomfort"  OR  "symptom*"  OR  pain ) )  AND (PUBYEAR > 1992) |
