# Supplementary material for: The associations of mobile touch screen device use with musculoskeletal symptoms and exposures: A systematic review
Source: PLoS One. 2017 Aug 7;12(8):e0181220. doi: 10.1371/journal.pone.0181220 (PMC5546699; doi:10.1371/journal.pone.0181220)
Supplement: S2 File — (DOCX) [file pone.0181220.s002.docx]

S2. Methodological quality assessment list

| 1. **Study purpose** | |
| --- | --- |
| + | A specific clearly stated purpose is well described and appropriate. |
| - | A specific clearly stated purpose is well described but not appropriate. |
| ? | Unclear or insufficient information. |
| 1. **Study design** | |
| + | Design used is well described and is appropriate to address the study purpose. |
| - | Design used is well described but not appropriate to address the study purpose. |
| ? | Unclear or insufficient information. |
| 1. **Study population** | |
| + | Definition and description (eligibility criteria, methods of selection and possible selection bias) of the subject group(s) involved in the study are well described and appropriate. |
| - | Definition and description (eligibility criteria, methods of selection and possible selection bias) of the subject group(s) involved in the study are well described but not appropriate. |
| ? | Unclear or insufficient information. |
| 1. **Musculoskeletal exposures and/or physiological responses** | |
| + | Musculoskeletal exposure and/or physiological responses variable(s), e.g., physical load during the test conditions measured by electromyography/kinematics, duration/frequency of device use etc., are well described and appropriately collected using standardized method(s) of acceptable quality (reliability and validity). |
| - | Musculoskeletal exposure and/or physiological responses variable(s), e.g. physical load during the test conditions measured by electromyography/kinematics, duration/ frequency of device use etc., are well described but not appropriately collected using standardized method(s) of acceptable quality (reliability and validity). |
| ? | Unclear or insufficient information. |
| 1. **Musculoskeletal symptoms (if applicable)** | |
| + | Musculoskeletal symptoms, i.e. pain, discomfort, soreness, injuries are well described and collected using appropriate standardized method(s) of acceptable quality (reliability and validity). |
| - | Musculoskeletal symptoms, i.e. pain, discomfort, soreness, injuries are well described but not collected using appropriate standardized method(s) of acceptable quality (reliability and validity). |
| ? | Unclear or insufficient information. |
| 1. **Statistical analyses** | |
| + | The statistical analyses applied are well described and appropriate for the outcome studied. |
| - | The statistical analyses applied are well described but not appropriate for the outcome studied. |
| ? | Unclear or insufficient information. |
| 1. **Results** | |
| + | Results are well described and appropriately reported in sufficient details. |
| - | Results are well described but not appropriately reported in sufficient details. |
| ? | Unclear or insufficient information. |

Note: (+) positive; (-) negative; (?) unclear, i.e. insufficient information
