# Supplementary material for: The associations of mobile touch screen device use with musculoskeletal symptoms and exposures: A systematic review
Source: PLoS One. 2017 Aug 7;12(8):e0181220. doi: 10.1371/journal.pone.0181220 (PMC5546699; doi:10.1371/journal.pone.0181220)
Supplement: S3 File — (DOCX) [file pone.0181220.s003.docx]

| S3. Summary of included cross-sectional studies (MTSD use and musculoskeletal symptoms)   \| **Author (year)** \| **Study population** \| **Type of MTSD examined** \| **Study design and conditions** \| **Musculoskeletal symptoms measurement** \| **Musculoskeletal symptoms**  **results** \| \| --- \| --- \| --- \| --- \| --- \| --- \| \| **Chiang and Liu (2016) [33]**  *Study 1*  *(an experimental laboratory study was also conducted (study 2) and is listed in S8)* \| **n** = 80  **Age:** > 20 years  **Gender:** 26 males, 54 females  **Other specific:** College students in Taiwan \| Tablet computer \| **Design:**  Cross-sectional study (study 1)  Questionnaire conducted and tablet tilt angle set up on tablet cases for various tasks by participants measured in the lab  **Conditions:** NA \| 1. **Type of symptoms:**   Discomfort of the neck, right and left shoulder, waist, back and distal upper extremities (i.e. wrist, upper arm, forearm)  **Measurement method:**  Questionnaire on discomfort/pain due to tablet use, indicating the body part(s) and pain using a visual analogue scale (VAS) from “no pain” to “worst pain imaginable” with the length of 10 cm  **Variable(s):**   - Discomfort and pain score \| - 55% (44 out of 80 participants) reported discomfort after using a tablet - Neck (37.5%) and shoulders (30% right shoulder, 26.3% left shoulder) were the most frequent areas of discomfort reported; - 7.5% (6 out of 80 participants) reported discomfort in the back after using a tablet - Mean VAS scores after using a tablet by participants who reported discomfort: back 5.6 (1.49); neck 4.91 (2.35), right shoulder 4.98 (2.51), left shoulder 5.26 (2.39) - No statistically significant differences were present between discomfort and reported daily tablet usage - Significantly more participants who tended to play games reported discomfort after playing game on tablet; no differences with the other tasks \| \| **Kim and Kim (2015) [25]** \| **n** = 292  (300 completed survey, 8 excluded due to incomplete data)  **Age:** 21.4 (1.6) years  **Gender:** -  **Other specific:** Dental hygiene students in South Korea \| Smartphone \| **Design:**  Cross-sectional study  Questionnaire on screen size, purposes, location, daily usage and postures of smartphone use  **Conditions:** NA \| 1. **Type of symptoms:**   Pain in the neck, shoulder, waist, distal upper extremities (i.e. hands, wrists, fingers), legs and feet  **Measurement method:** Questionnaire on symptoms in the above-mentioned body regions  **Variable(s):**   - Pain at body regions \| - Neck and shoulder were the most common areas of pain reported by participants (neck 55.8%, shoulder 54.8%, arms 19.2%, hands 19.2%, wrists 27.1%, fingers 19.9%, waist 29.8%, 9.6% legs and feet) - Significant positive correlation between pain in the waist region and screen size (r=0.129), no significant correlations of screen size with pain in any other body region - Significant negative correlation between pain in the legs and feet and daily usage (r= -0.127), no significant correlations of daily usage with pain in any other body region - Only descriptive information was available for the following results (no statistical analyses were performed): - There was a higher prevalence of pain in those who use smartphones whilst sitting and lying on their back compared to those who adopted other postures (standing, lying on the face or others); - There was a higher prevalence of pain in those who use smartphones for searching internet and chatting compared to those who use smartphone for other purposes; - There was a higher prevalence of pain in those who used a smartphone for >2 hours daily compared to those who used a smartphone for <2 hours daily \| \| **Shan et al (2013) [24]** \| **n** = 3016  **Age** = 15-19 years  **Gender:**  1,460 males, 1,556 females  **Other specific:** High school students from 30 randomly selected schools in Shanghai, China; 83.8% response rate \| Tablet computer \| **Design:**  Cross-sectional study  Questionnaire on:   - Daily tablet usage (<1hour/ 1-1.5 hours/ 1.5-2hours/ >2 hours) - Posture during tablet use (standing/ lying/ semi-reclining/ sitting) - Eye-to-screen distance while using tablet   **Conditions:** NA \| 1. **Type of symptoms:**   Neck/shoulder and low back pain  **Measurement method:**  Questionnaire on discomfort in the neck/shoulder and lower back and its frequency during the past 6 months  (“almost never” (<1/month); “occasionally” (1-3x/month); “often” (1-3x/week); “always” (>3x/week); frequency of “often” and “always” denoted as presence of pain)  **Variable(s):**   - Neck/shoulder pain - Low back pain \| - Prevalence of neck/shoulder pain: 40.8% - Females had a significantly higher odds of neck/shoulder pain than males (OR 1.293, 95% CI 1.108-1.509) - Tablet use was significantly associated with neck/shoulder pain (OR 1.311, 95% CI 1.117-1.538) - No significant association of tablet daily usage, posture during tablet use and eye-to-screen distance while using tablet with neck/shoulder pain - Prevalence of low back pain: 33.1% - Females had a significantly higher odds of low back pain than males (OR 1.296, 95% CI 1.102-1.523) - No significant association of tablet use, tablet daily usage, posture during tablet use and eye-to-screen distance while using tablet with low back pain \| \| **Sommerich et al (2007) [23]** \| **n** = 77  **Age:** -  **Gender:** 19 males, 58 females  **Other specific:** Grade 11 or 12 students from a school in USA (73% completion rate) \| Tablet computer \| **Design:**  Cross-sectional study  Questionnaire on:   - Frequency and duration of tablet use - Frequency of awkward postures when using tablet - Moving/carrying tablet - Tablet temporal usage patterns - Activities on the tablet - Tools and peripheral devices for tablet (e.g. external key board)   **Conditions:** NA \| 1. **Type of symptoms:**   Discomfort in the neck, shoulder, distal upper extremities (i.e. forearm/elbow, hand/wrist), upper back, lower back, buttocks and legs/feet, eyes and head (headache)  **Measurement method:**  Questionnaire on discomfort associated with tablet use and moving/carrying tablet, its body region and frequency (never/ rarely/ sometimes/ quite often/ almost always)  **Variable(s):**   - Discomfort score \| - 50% or more reported discomfort in the eyes, neck, head (headache), right hand/wrist and upper back, upper and lower back - 30-40% reported discomfort in the neck, lower back and eyes at least “sometimes”, <10% reported “quite often” or “almost always” - Shoulder was the most common region reported (21.6%) for discomfort when moving/carrying tablet - Frequency of awkward postures when using tablet use was significantly correlated with overall discomfort scores (r=0.40), and discomfort in the right shoulder, neck, upper back, low back, buttocks, eyes and head (headache) - No significant correlation between discomfort and duration of continuous tablet use in sitting, moving/carrying tablet, and other aspects of tablet use were shown \| |
| --- | --- | --- | --- | --- | --- | --- | --- | --- | --- | --- | --- | --- | --- | --- | --- | --- | --- | --- | --- | --- | --- | --- | --- | --- | --- | --- | --- | --- | --- | --- |
