# Supplementary material for: The associations of mobile touch screen device use with musculoskeletal symptoms and exposures: A systematic review
Source: PLoS One. 2017 Aug 7;12(8):e0181220. doi: 10.1371/journal.pone.0181220 (PMC5546699; doi:10.1371/journal.pone.0181220)
Supplement: S4 File — (DOCX) [file pone.0181220.s004.docx]

| S4. Summary of included case-control laboratory studies (MTSD use and musculoskeletal symptoms)   \| **Author (year)** \| **Study population** \| **Type of MTSD examined** \| **Study design and conditions** \| **Musculoskeletal symptoms measurement** \| **Musculoskeletal symptoms**  **results** \| \| --- \| --- \| --- \| --- \| --- \| --- \| \| **Kee et al (2016) [44]** \| **n** = 100  **Age:** Normal group 16.9 (1.6) years; addicted group 17.0 (2.0) years  **Gender:** 28 males, 72 females  **Other specific:** Teenagers and patients recruited from a hospital department in South Korea \| Smartphone \| **Design:**  Case-control laboratory study  **Conditions:**  All participants have temporomandibular disorders, and were (based on smartphone addiction scale) grouped into:   - Normal group - Addicted group   Further categorised into sub-diagnosis of muscular, joint or mixed problems \| 1. **Type of symptoms:**   Temporomandibular disorders  **Measurement method:**  Based on complaints, clinical histories, x-rays of temporomandibular joint and lateral cephalometric and clinical examinations; diagnosis further subdivided into joint, muscular or mixed problems  **Variable(s):**   - Sub-diagnosis of temporomandibular disorders \| - Normal group had 36 participants with joint problems, 9 with muscular problems, and 5 with mixed problems - Addicted group had 22 participants with joint problems, 21 with muscular problems, and 7 with mixed problems \| \| **Lee et al (2012) [51]** \| **n** = 125  **Age:** 21.4 (2.0) years  **Gender:** 32 males, 93 females  **Other specific:** Students from university in South Korea, and no wrist symptoms or injuries \| Smartphone \| **Design:**  Case-control laboratory study  Based on Nomophobia syndrome questionnaire, participants were grouped into 3 groups for each of the categories below:   - Smartphone addiction degree - Daily usage duration - Continuous using time - Total periods of use   **Conditions:** NA \| 1. **Type of symptoms:**   Hypesthesia or dysesthesia in areas controlled by the median nerve  **Measurement method:**  Phalen’s and reverse Phalen’s tests  **Variable(s):**   - Hypesthesia or dysesthesia \| - There was a significantly shorter time for paraesthesia to appear in the smartphone addiction degree group of ≥30 points, than in the lesser addicted groups (with 20-29 and ≤19 points, respectively) - There was a significantly shorter time for paraesthesia to appear in the long continuous smartphone usage group of (≥60 mins), than in groups with shorter usage (<60mins and <30mins, respectively) - There was significantly shorter time for paraesthesia to appear in a group with longer duration of smartphone usage (≥8 hours), than in groups with shorter duration of usage (3-7 hours and ≤3 hours, respectively) - There were no significant differences in hypesthesia or dysesthesia among groups with different total periods of use (≤12, 13-24 or ≥25 months, respectively) \| \| **Inal et al (2015) [41]** \| **n** = 102  **Age:** 18 to 23 years  **Gender:** 30 males, 72 females  **Other specific:** Students from a university in Turkey, 66 of them had a habit of one handed smartphone use \| Smartphone \| **Design:**  Case-control laboratory study  Based on a smartphone addiction scale, participants were grouped into (with the median score of 84 used to classify high and low users):   - High users (≥84 score), vs - Low users (≤84 score) vs - Non-users   **Conditions:** NA \| 1. **Type of symptoms:**   Pain in the hand  **Measurement method:**  Visual analogue scale (VAS) (0 to 10cm) for pain in the dominant hand at rest and during movement (in the previous week)  **Variable(s):**   - Pain score \| - Pain during movement was significantly higher in high smartphone users (1.06 (2.18)) than in low smartphone users (0.06 (0.34)) - There were no difference in pain at rest among non-smartphone users, low and high smartphone users - Significant correlations between pain at rest (r=0.272) and during movement (r=0.345) with smartphone addiction scale scores \| \| **Xie et al (2016) [21]** \| **n** = 40  **Age:** 23.9 (3.2) years  **Gender:** 16 males, 24 females  **Other specific:** Right handed participants, recruited from universities in Hong Kong \| Smartphone \| **Design:**  Case-control laboratory study  Based on questionnaires responses, grouped into:   - Case group (n=20, with neck/shoulder discomfort) vs - Control group (n=20, without neck/shoulder discomfort)   **Conditions:**  While sitting for 10 minutes, participants performed:   - Two-handed texting (both thumbs) at chest level vs - One-handed texting (right thumb) at chest level vs - Two-handed typing on desktop computer   **Task:**  Typing \| 1. **Type of symptoms:**   Discomfort in the neck, and dominant shoulder, upper back and distal upper extremity (i.e. elbow, wrist/hand and thumb/fingers)  **Measurement method:**  Scores from numeric rating scale (0 to 10) for all the above body regions summed up  **Variable(s):**   - Discomfort score \| - Discomfort scores were significantly higher in the case group than in the control group for one and two-handed texting on smartphone and two-handed typing on desktop - There were no significant differences in discomfort among smartphone texting and desktop typing tasks within each group \| |
| --- | --- | --- | --- | --- | --- | --- | --- | --- | --- | --- | --- | --- | --- | --- | --- | --- | --- | --- | --- | --- | --- | --- | --- | --- | --- | --- | --- | --- | --- | --- |
