# Supplementary material for: The associations of mobile touch screen device use with musculoskeletal symptoms and exposures: A systematic review
Source: PLoS One. 2017 Aug 7;12(8):e0181220. doi: 10.1371/journal.pone.0181220 (PMC5546699; doi:10.1371/journal.pone.0181220)
Supplement: S5 File — (DOCX) [file pone.0181220.s005.docx]

| S5. Summary of included experimental laboratory studies (MTSD use and musculoskeletal symptoms) | | | | | |
| --- | --- | --- | --- | --- | --- |
| **Author (year)** | **Study population** | **Type of**  **MTSD**  **examined** | **Study design and conditions** | **Musculoskeletal symptoms measurement** | **Musculoskeletal symptoms**  **results** |
| **Ahn et al (2016) [36]** | **n** = 26  **Age:** 24.7 (3.4) years  **Gender:** 19 males, 7 females  **Other specific:** College students in South Korea | Smartphone (mock up device) | **Design:**  Experimental laboratory study  **Conditions:**  Using the smartphone with one hand (preferred hand) for 50 minutes, tasks were executed with:   - Touch screen flat surface - Less curved surface (400R) - More curved surface (100R)   Participants were divided into groups according to:   - Hand size (small/ large hands) - Hand shape (long palms/ long fingers)   **Task:**  Tapping and dragging | 1. **Type of symptoms:**   Comfort in the hand  **Measurement method:**  Subjective rating on a 100 point scale  **Variable(s):**   - Comfort rating | - Comfort was significantly higher when using the less curved surface smartphone compared to the flat surface smartphone; no differences between more curved surface and less curved or flat surface were shown - No significant effect of hand size and hand shape on comfort were reported - A significant interaction effect of hand size and shape on comfort was found with lower comfort in participants with large hands long fingers compared to those with large hands long palms |
| **Albin and Mcloone (2014) [37]** | **n** = 10  **Age:** -  **Gender:** -  **Other specific:** Right handed university students from the USA | Tablet computer | **Design:**  Experimental laboratory study  **Conditions:**  Using the tablet in portrait orientation while sitting, the following tilt angles were varied:   - 0°, 30°, 45°, 60° and a self-chosen angle   **Task:**  Reading and tapping | 1. **Type of symptoms:**   Comfort in the hands and arms  **Measurement method:**  Likert scale 1 (strongly agree) to 7 (strongly disagree)  **Variable(s):**   - Comfort score | - The highest comfort scores were seen with self-selected tilt angle of 34° (19)) - The lowest comfort scores were seen with 60° tilt angle |
| **Chiu et al (2015) [35]** | **n** = 30  **Age:** 23.5 (2.8) years  **Gender:** 16 males,  14 females  **Other specific:** Right handed participants | Tablet computer | **Design:**  Experimental laboratory study  **Conditions:**  With a tablet on a desk and the participant sitting, tablet tilt angles were varied:   - 22.5° vs 45° vs 67.5°   **Task:**  Movie watching vs game playing (for 15 minutes) | 1. **Type of symptoms:**   Comfort in the neck, shoulder, upper arm, forearm, wrist and finger (right side)  **Measurement method:**  Visual analogue scale (VAS) (0=extreme discomfort, 10=extreme comfort)  **Variable(s):**   - Comfort score | - A significant main effect of tilt angle on perceived neck comfort was shown, but no differences among tilt angles were found in the post-hoc analysis - No main effect of tilt angle were found for any of the other body regions - Significantly lower comfort in the shoulder, upper arm, forearm, wrist and finger (but not in the neck) was found during game playing compared to when watching movie |
| **Kim et al (2014) [18]** | **n** = 19  **Age:** 24.3 (6.4) years  **Gender:** 10 males, 9 females  **Other specific:** Participants were experienced touch typists, 17 participants were right handed | Virtual (touch screen) keyboard on a laptop | **Design:**  Experimental laboratory study  **Conditions:**  While sitting for 10 minutes, tasks were performed on:   - A virtual keyboard on laptop vs - A physical keyboard on laptop vs - A desktop computer keyboard   **Task:**  Typing | 1. **Type of symptoms:**   Comfort in the hand/wrist and arm/shoulder  **Measurement method:**  Modified Likert scale (1=least comfortable; 7=most comfortable) adapted from ISO keyboard comfort questionnaire  **Variable(s):**   - Comfort score at hand/wrist - Comfort score at arm/shoulder | - Significant lower comfort when typing on a virtual keyboard (2.9 (1.7)) than on a physical keyboard on a laptop (5.4 (1.1)) or desktop (5.4 (1.2)) were found - Significant lower comfort when using a virtual keyboard (3.4 (1.7)) compared to a physical keyboard on a laptop (5.3 (1.2)) or a desktop (4.9 (1.2)) were found |
| **Kim et al (2014) [47]** | **n** = 21 (data analysed for 19 only)  **Age:** 24.5 years (18 to 49 years)  **Gender:**  12 males, 9 females  **Other specific:** Participants were experienced USA touch typists, 19 participants were right handed | Notebook computer with virtual (touch screen) keyboard | **Design:**  Experimental laboratory study  **Conditions:**  While sitting, 2x5minute tasks were performed on 4 virtual keyboard with different key sizes:   - 13, 16, 19, and 22mm (width and height), and vertical center-to-center key spacing of 15, 18, 21, and 24mm   **Task**:  Typing | 1. **Type of symptoms:**   Comfort in the arm/shoulder and hand/wrist  **Measurement method:**  7-point Likert scales adapted from the ISO keyboard comfort questionnaire  **Variable(s):**   - Comfort score | - Arm/shoulder and hand/wrist comfort generally increased with increasing key sizes - Smallest key size (13mm) was associated with significantly lower comfort in the hand/wrist (2.3 (0.2)) and arm/shoulder (2.7 (0.3)) compared to the larger key sizes (19 & 22mm); - No differences on comfort across the other 3 larger keyboard key sizes (16, 19, & 22mm) were seen |
| **Lin et al (2015) [54]** | **n** = 18  **Age:** males 24.8 (3.5); females 23.1 (0.9) years  **Gender:** 9 males, 9 females  **Other specific:** All were right handed | Tablet computer | **Design:**  Experimental laboratory study  **Conditions:**  Tablet use on different workstations:   - Desk (flat on desk) vs - Lap (flat on lap) vs - Bed (inclined sitting on bed, tablet on lap)   Using different virtual keyboard designs:   - Standard vs - Wide vs - Split   **Task:**  Typing | 1. **Type of symptoms:**   Discomfort in the neck, shoulder, upper back, lower back, distal upper extremity (i.e. wrists, arms) and buttocks  **Measurement method:**  Borg’s category rating scale and body map with perceived discomfort  **Variable(s):**   - Discomfort score | - Discomfort in all the body regions significantly increased with time - Discomfort in the shoulder, neck, upper and lower back and buttocks was significantly higher with using the tablet on the lap compared to on a desk or bed - In general, discomfort in the wrists and arms was significantly higher with tablet on the lap compared to on a desk - In general, discomfort in all body regions was significantly higher while using it on a bed compared to on a desk - In general, discomfort in the shoulder, wrists and arms was significantly lower while using a split keyboard compared to a standard keyboard - No differences in neck, upper lower back and buttocks discomfort were shown among different keyboard designs |
| **Shin and Kim (2014) [59]** | **n** = 15  **Age**: 26.1 (5.7) years  **Gender**: -  **Other specific:** Participants had no history of neck pain or spinal injuries in the past year | Smartphone | **Design:**  Experimental laboratory study  **Conditions:**  Smartphone use with two-handed hold for 15 minutes, while sitting with:   - A neutral neck posture with smartphone on a desk vs - A flexed neck with smartphone on the lap   **Task:**  Free use of applications of choice | 1. **Type of symptoms:**   Neck pain  **Measurement method:**  Visual analogue scale (VAS) ranging from 0 to 10  **Variable(s):**   - VAS score | - Neck pain Increased from 0 to 1.7 during the condition of smartphone use with a neutral neck posture on desk - Neck pain increased from 0 to 5.2 during the condition of smartphone use on the lap with a flexed neck posture (no results of a statistical test were reported) |
| **Trudeau et al (2013) [62]** | **n** = 12  **Age:** 30.0 (5.1) years  **Gender:**  6 males, 6 females  **Other specific:** All participants were right handed | Tablet computer | **Design:**  Experimental laboratory study  **Conditions:**  Tablet use with two handed hold while sitting for 2 minutes, during 11 conditions in which the following independent variables were varied:   - Tablet orientation (portrait/ landscape) - Keyboard layouts (standard/ split) - Keyboard locations (top/ middle/ bottom)   **Task:**  Typing | 1. **Type of symptoms:**   Discomfort of the neck, back and/or distal upper extremities (i.e. arm, hand, thumbs)  **Measurement method:**  Visual analogue scale (VAS) ranging from 1 to 10 for physical discomfort or pain experienced in any part of the above body regions  **Variable(s):**   - Discomfort score (one overall score reported for each condition) | - Significantly higher discomfort was reported when using a tablet in landscape orientation (3.6 (0.6)) compared to in portrait orientation (2.9 (0.6)) - Significantly higher discomfort was reported when typing on a tablet in standard keyboard layout (3.7 (0.6)) compared to when using a split keyboard layout (2.8 (0.6)) when tablet was in landscape orientation; however, no significant differences regarding keyboard layout were seen in tablet portrait orientation - No differences among different keyboard locations were shown |
