# Supplementary material for: The associations of mobile touch screen device use with musculoskeletal symptoms and exposures: A systematic review
Source: PLoS One. 2017 Aug 7;12(8):e0181220. doi: 10.1371/journal.pone.0181220 (PMC5546699; doi:10.1371/journal.pone.0181220)
Supplement: S6 File — (DOCX) [file pone.0181220.s006.docx]

S6. Summary of included cross-sectional studies (MTSD use and musculoskeletal exposures)

| **Author** | **Study population** | **Type of MTSD examined** | **Study design and**  **conditions** | **Musculoskeletal exposures measurement** | **Musculoskeletal exposures**  **results** |
| --- | --- | --- | --- | --- | --- |
| **Guan et al (2016) [34]** | **n** = 429  **Age:** 19.8 (2.6) years  **Gender:** 219 males,  210 females  **Other specific:** University students in Shanghai, China | Smartphone | **Design:**  Cross-sectional study  *(an experimental laboratory study was also conducted and is listed in S8)*  **Conditions:** NA | 1. **Type of exposures:**   Mobile phone usage  **Measurement method:**  Questionnaire on type, years of usage and daily usage of smartphone  **Variable(s):**   - Mobile phone usage | - 97.7% (419/429) used a smartphone, 1.2% (5/429) used a non-smartphone - Years of mobile phone usage:   ≤1 year: 91/429;  >1 and ≤ 3 years: 111/429;  >3 and ≤6 years: 148/429;  >6 years: 78/429   - Daily mobile phone usage:   ≤ 1 hour: 36/429;  >1 and ≤ 3 hours: 193/429;  >3 and ≤ 5 hours: 123/429;  >5 hours: 72/429 |
| **Liang and Hwang (2016) [53]** | **n** = 1230  **Age:** <20 to >60 years (estimated)  **Gender:** 454 males, 776 females  **Other specific:** Passengers in Taipei, Taiwan metro train who were using mobile phones (sample of 400 observation trips) | Smartphone | **Design:**  Cross-sectional study  **Conditions**: NA | 1. **Type of exposures:**   Arms, trunk and leg posture  **Measurement method:**  Observation checklist adapted from a previous study for standing and sitting  **Variable(s):**   - Arms, trunk and leg posture  1. **Type of exposures:**   Screen operating styles **Measurement method:**  Categorized into 4 groups:   - Both hands hold and operate - One hand hold and operate - Different hands hold and operate - Others (unclassified)   **Variable(s):**   - Screen operating styles | - Among sitting users, the most frequently observed posture was trunk against the backrest, wrist/forearm supported and both feet on the floor (31.6%), followed by a similar posture but free from armrests (26.6%), and trunk against backrest, elbow supported and legs free, both feet on floor (8.9%) - Among standing users, the most frequently observed posture was trunk against a wall/pole, arms free from support and both feet on the floor (30.8%), followed by a similar posture, but with the trunk free from support (23%), and back against wall/pole, arms free from support and single-foot stance (16.5%) - The majority of passengers (~78%) used one hand rather than two hands to hold their mobile phones; using the left hand only was more common than using the right hand - Most common style is different hands to hold and operate (39.5%), followed by one hand (30%), both hands (22%) and others (unclassified) (8.5%) - The majority of standing users used one hand to hold and operate (45.8%), while the majority of the sitting users used a different hand for holding (45.5%) versus operating (22%) - **For users sitting**, postures of trunk against backrest, feet on floor and with or without arm support most commonly observed across the 4 groups - **For users standing**, both hands and different hands group used a high proportion of postures - trunk supported or not, arm free and both feet on the floor; one hand group most frequently used postures - trunk unsupported, holding a pole/handstrap and both feet on floor |
