# Supplementary material for: The associations of mobile touch screen device use with musculoskeletal symptoms and exposures: A systematic review
Source: PLoS One. 2017 Aug 7;12(8):e0181220. doi: 10.1371/journal.pone.0181220 (PMC5546699; doi:10.1371/journal.pone.0181220)
Supplement: S7 File — (DOCX) [file pone.0181220.s007.docx]

S7. Summary of included case-control laboratory studies (MTSD use and musculoskeletal exposures)

| **Author** | **Study population** | **Type of MTSD examined** | **Study design and**  **conditions** | **Musculoskeletal exposures measurement** | **Musculoskeletal exposures**  **results** |
| --- | --- | --- | --- | --- | --- |
| **Jung et al (2016) [43]** | **n** = 50  **Age:** 21.0 (2.4) years  **Gender:** -  **Other specific:**  Participants were recruited from a university in South Korea | Smartphone | **Design:**  Case-control laboratory study  Categorised into 2 groups according to reported smartphone usage:   - Low users   (n=50; <4 hours/day)   - High users   (n=50; >4 hours/day)  **Conditions:** NA | 1. **Type of exposures:**   Neck postures  **Measurement method:**  Lateral image taken in usual standing posture using a digital camera  **Variable(s):**   - Neck flexion WRT horizontal  1. **Type of exposures:**   Shoulder postures  **Measurement method:**  Scapular index calculated using body landmarks measured via measuring tape in standing  **Variable(s):**   - Scapular index | - Significantly lower in smartphone high users (53.0° (6.3)) than low users (54.5° (4.2)) - Significantly lower (i.e. greater rounded shoulder postures) in smartphone high users (65.5 (6.5)) than low users (67.5 (4.2)) |
| **Kee et al (2016) [44]** | **n** = 100  **Age:** normal group 16.9 (1.6) years; addicted group 17.0 (2.0) years  **Gender:**  28 males, 72 females  **Other specific:** All teenagers and patients recruited from a hospital department in South Korea | Smartphone | **Design:**  Case-control laboratory study  **Conditions:**  All participants have temporomandibular disorders; based on smartphone addiction scale (short version), grouped into:   - Normal group - Addicted group   Further categorised into sub-diagnosis of muscular, joint or mixed problems | 1. **Type of exposures:**   Neck posture  **Measurement** **method:**  Lateral skull radiograph assessment in resting posture  **Variable(s):**   - Cervical lordosis angle and craniocervical angle  1. **Type of exposures:**   Neck ROM  **Measurement method:**  Cervical ROM instrument on head  **Variable(s):**   - Cervical flexion (during habitual sitting posture when using smartphone) - Cervical flexion, cervical extension, right and left lateral flexion, right and left rotation, and cervical protrusion (when not using any device) | - No differences in cervical lordosis and craniocervical angles between normal and addicted group - Cervical flexion when using smartphone in sitting significantly higher in the addicted group (28.0° (13.0)) than in the normal group (12.9° (10.6)) - All neck ROM variables were significantly lower in the addicted group than in the normal group, except for cervical protrusion where no differences were detected - Significant differences in all neck ROM measurements between the addicted and normal groups with temporomandibular disorder muscular problems, except for lateral flexion - Significant differences in all neck ROM measurements between the addicted and normal groups with joint problems, except for right lateral flexion and cervical flexion - No difference in neck ROM between the addicted and normal groups with mixed problem, except for cervical flexion |
| **Kim (2015) [45]** | **n** = 27  **Age:** control group 20.6 (1.6) years; mild neck pain group 20.6 (1.5) years  **Gender:** 12 males, 15 females  **Other specific:** All experienced cervical symptoms while using a smartphone within the last year; participants were recruited from a university in South Korea | Smartphone | **Design:**  Case-control laboratory study  Based on neck disability index (NDI) score, participants were grouped into:   - Mild neck pain group (n=13, score >8) - Control group (n=14, score ≤8)   **Conditions:**  Smartphone use for 3x5 minutes  **Task:**  Free use of text messaging and internet browsing | 1. **Type of exposures:**   Neck posture  **Measurement method:**  Using an ultrasound motion analysis system  **Variable(s):**  Mean angles at 100s, 200s and 300s:   - Upper and lower cervical flexion | - Both upper and lower cervical flexion angles were significantly higher in the mild neck pain group than in the control group during smartphone use - No differences for in neck flexion over time between the mild neck pain and control group - Significant variations in lower cervical flexion angles over time in mild neck pain group than in the control group were seen; but not in upper cervical flexion |
| **Park et al (2015) [56]** | **n =** 20  **Age:** 23.3 (2.3) years  **Gender:** -  **Other specific:** Recruited from a university in South Korea | Smartphone | **Design:**  Case-control laboratory study  **Conditions:**  According to scores on the smartphone addiction scale, participants were grouped into:   - Heavy user group (n=10, scores 45.1 (3.6)) - Control group (n=10, scores 26.5 (4.5)) | 1. **Type of exposures:**   Head and neck posture  **Measurement method:**  Posture were observed by a physical therapist with a plumb line and analysed using Adobe Acrobat software (no information on the exact method)  **Variable(s):**   - Craniovertebral angle - Head position angle | - No differences in craniovertebral angle between heavy smartphone user and control group - Significantly lower head position angle in heavy user group (34.9° (5.4)) than in the control group (39.7° (3.0)) |
| **Xie et al (2016) [21]** | **n** = 40  **Age:** 23.9 (3.2) years  **Gender:** 16 males, 24 females  **Other specific:** All were right handed and recruited from universities in Hong Kong | Smartphone | **Design:**  Case-control laboratory study  Based on questionnaires responses, grouped into:   - Case group (n=20; with neck shoulder discomfort) - Control group (n=20; no discomfort)   **Conditions:**  In sitting for 10 minutes:   - Two-handed texting (both thumbs) at chest level vs - One-handed texting (right thumb) at chest level vs - Two-handed typing on desktop computer   **Task:**  Typing | 1. **Type of exposures:**   Neck, shoulder, wrist, fingers and thumb muscle activity  **Measurement method:**  EMG on CES, UT, LT, ECR, ED, FDS and APB  **Variable(s):**  10th, 50th (median) and 90th percentile APDF of:   - CES (neck) - UT and LT (neck/shoulder) - ECR, ED, FDS and APB (wrist, fingers and thumb) | - No differences between case and control group, but a trend of consistently higher muscle activity in the case group compared to the control group were shown in all tasks - CES activity was significantly higher (10^th^ percentile) during two-handed smartphone texting than during desktop typing in both groups - No differences between one-handed and two-handed texting for both groups - UT median activity was significantly higher in the case group than in the control group; no differences in LT activity between case group and control group were seen - UT and LT median activity was significantly lower during two-handed smartphone texting than during desktop typing - Higher UT and LT activity (but non-significant differences) in one-handed compared to two-handed smartphone texting for both groups were seen - No differences between case group and control group for all the 4 distal muscles - ECR and ED median muscle activity was significantly lower, APB activity was significantly higher during two-handed smartphone texting than during desktop typing for both groups - All median muscle activity of 4 distal muscles was significantly lower during two-handed than during one-handed smartphone texting for both groups |
| **Xiong and Muraki (2016) [66]** | **n** = 48  **Age:** youth 23.6 (1.8) years; elderly 67.5 (3.7) years  **Gender:** 24 males, 24 females | Smartphone | **Design:**  Case-control laboratory study  Grouped into:   - Shorter thumb vs longer thumb group - Youth (n=24) vs elderly group (n=24)   **Conditions:**  Tapping self-identified points (far and close) on the screen while sitting, with the right hand holding two phone types:   - Smaller touch screen size vs - Larger touch screen size | 1. **Type of exposures:**   Thumb coverage on screen  **Measurement method:**  Tapping points recorded via pre-scale pressure imaging, connected together to obtain coverage area and intersection point for centre of gravity  **Variable(s):**   - Thumb coverage area - Centre of gravity in thumb coverage area | - Thumb coverage area was significantly greater for those with longer thumbs than those with shorter thumbs in both screen sizes - Thumb coverage area increased significantly with larger screen size compared to smaller screen size, but it did not increase at the same ratio as the increase in screen size - Elderly and longer thumb groups tend to have more   unreachable space (i.e. how far the thumbs can reach) at the right side and bottom of touch screens, compared to the youth and shorter thumb groups |

***Abbreviated terms:*** *APB: abductor pollicis brevis; CES: cervical erector spinae; ECR: extensor carpi radialis; ED: extensor digitorium; FDS: flexor digitorium superficialis; LT: lower trapezius;*

*ROM: range of motion; UT: upper trapezius; WRT: with respect to*
