# Supplementary material for: The associations of mobile touch screen device use with musculoskeletal symptoms and exposures: A systematic review
Source: PLoS One. 2017 Aug 7;12(8):e0181220. doi: 10.1371/journal.pone.0181220 (PMC5546699; doi:10.1371/journal.pone.0181220)
Supplement: S8 File — (DOCX) [file pone.0181220.s008.docx]

S8. Summary of included experimental laboratory studies (MTSD use and musculoskeletal exposures)

| **Author** | **Study population** | **Type of MTSD examined** | **Study design and**  **conditions** | **Musculoskeletal exposure measurement** | **Musculoskeletal exposures**  **results** |
| --- | --- | --- | --- | --- | --- |
| **Ahn et al (2016) [36]** | **n** = 26  **Age:** 24.7 (3.4) years  **Gender:** 19 males, 7 females  **Other specific:** College students in South Korea | Smartphone (mock-up device) | **Design:**  Experimental laboratory study  **Conditions:**  Using one hand (preferred side) to operate a smartphone for 50 minutes in three different conditions/participant groups:   - Screen surface (flat/ less curved (400R)/ more curved (100R)) - Hand size (small/ large hands) - Hand shape (long palms/ long fingers)   **Task:**  Tapping and dragging | 1. **Type of exposures:**   Fingers and thumb muscle activity  **Measurement method:**  EMG on FDS, ADM, OP, FPL  **Variable(s):**  Mean muscle activity of:   - FDS, ADM, OP and FPL (fingers and thumb) | - No difference in muscle activity for all muscles among different surface curvatures were shown - Significant interaction effect of hand size and shape on muscle activity; ADM, OP, and FPL activity was higher in participants with small hands compared to large hands, while FPL activity was higher in participants with long fingers compared to long palms |
| **Albin and Mcloone (2014) [37]** | **n** = 10  **Age:** -  **Gender:** -  **Other specific:** Right handed students from a university in the USA | Tablet computer | **Design:**  Experimental laboratory study  **Conditions:**  A tablet was used in portrait orientation while sitting with tablet tilt angles varying in:   - 0° - 30° - 45° - 60° - Self-chosen angle   **Task:**  Reading and tapping | 1. **Type of exposures:**   Head, neck, forearm and wrist posture  **Measurement method:**  Video analysis for head and neck, goniometer and torsiometer on forearm and wrist  **Variable(s):**   - Head/neck flexion - Forearm rotation, wrist flexion and lateral deviation | - Head/neck flexion decreased significantly with increasing tablet tilt angles (35^°^ (8.9) head/neck flexion at 0° tablet tilt; 24° (4.5) head/neck flexion at 30° tilt; 22° (6.2) at 45° tilt; 18° (3.8) head/neck flexion at 60° tilt) - No significant differences between tablet tilt angles for forearm or wrist postures were seen |
| **Billinghurst and Vu (2015) [38]** | **n** = 20  **Age:** psychologists 30.2 (6.8) years; engineers 30.1 (9.5) years  **Gender:** 19 males, 7 females  **Other specific:** All were right handed, recruited from a research centre in the USA; psychologists (n=10), engineers (n=10) | Smartphone and tablet computer | **Design:**  Experimental laboratory study  **Conditions:**   - Smartphone vs tablet - Device tilted (hold device with one hand and mounted on a stand) vs flat on a desk - Psychologists vs engineers   **Task:**  Participants performed common web browsing tasks of form submission, back and forward button, creating closing tabs, reloading page, return to home page, bookmarking and address bar | 1. **Type of exposures:**   Gestures performed  **Measurement method:**  Video cameras  **Variable(s):**   - Gestures performed | - The majority of gestures performed were made with one hand (dominant side), the index finger, and in a single motion - Gestures with five fingers were significantly more often performed on a tablet than on a smartphone - No differences in gestures between device tilted or flat on desk - Only small differences in hand gestures between psychologists and engineers were shown |
| **Chiang and Liu (2016) [33]**  *Study 2*  *(a cross-sectional study was also conducted (study 1) and is listed in S3)* | **n** = 30  **Age:** >20 years  **Gender:** 4 males, 26 females  **Other specific:** College students in Taiwan | Tablet computer | **Design:**  Experimental laboratory study (study 2)  **Conditions:**  Tablet in landscape orientation placed on a desk while sitting was operated (for 5 minutes) with the following tilt angles:   - 0° vs 45° vs 60°   **Task:**  Game playing vs reading | 1. **Type of exposures:**   Head and neck posture  **Measurement method:**  3D motion analysis system  **Variable(s):**   - Head flexion WRT vertical - Neck flexion WRT vertical | - Head flexion decreases with increasing tilt angle; head flexion was significantly higher at 0° (115.2° (1.7)) than at 45° tilt angle (103.9° (1.8)), and also higher at 45° than at 60° (98.1° (1.4)) - No difference in head flexion between game playing and reading tasks were found - Neck flexion decreases with increasing tilt angle; neck flexion was significantly higher at 0° (79.7° (1.8)) than at 45° tilt angle (71.4° (2.3)), and also higher at 45° than at 60° (64.3° (2.0)) - Neck flexion was significantly higher during game playing (73.6° (2.2)) than during reading tasks (70.0° (1.6)) |
| **Chiu et al (2015) [35]** | **n** = 30  **Age:** 23.5 (2.8) years  **Gender:** 16 males,  14 females  **Other specific:** All were right handed | Tablet computer | **Design:**  Experimental laboratory study  **Conditions:**  With the tablet on a desk and while sitting, the tablet was operated for 15 minutes in each of the following tilt angle conditions   - 22.5° vs 45° vs 67.5°   **Task:**  Movie watching vs game playing | 1. **Type of exposures:**   Neck/shoulder and fingers muscle activity  **Measurement method:**  EMG on UT, anterior and middle deltoid, FDS of dominant arm  **Variable(s):**  Mean muscle activity of:   - UT, anterior and middle deltoid (neck/shoulder) - FDS (fingers) | - Significantly higher muscle activity in UT, but lower in anterior deltoid during tablet use at lower tilt angle of 22.5°, than at higher tilt angles of 45° and 67.5°; there were no differences in middle deltoid muscle activity between different tilt angles - Significantly higher UT, anterior and middle deltoid muscle activity during game playing than during movie watching - Significantly higher FDS muscle activity during game playing than during movie watching; there were no differences between tilt angles |
| **Choi et al (2016) [39]** | **n** = 15  **Age:** 23.6 (2.4) years  **Gender:** 8 males,  7 females  **Other specific:** College students in South Korea | Smartphone | **Design:**  Experimental laboratory study  **Conditions:**  Continuous texting while sitting for 5 minutes with:   - Neutral neck (upper part of phone screen at eye level) - Middle neck bending (neck bent in comfortable manner) - Maximum neck bending   **Task:**  Typing | 1. **Type of exposures:**   Neck/shoulder muscle activity  **Measurement method:**  EMG on right and left splenius capitis and UT  **Variable(s):**  Mean muscle activity of:   - Splenius capitis and UT (neck/shoulder) | - No differences in all muscle activities between neutral, middle or maximum neck bending postures when texting on smartphone were seen |
| **Guan et al (2015) [40]** | **n** = 186  **Age:** 21.0 years (17 to 31 years)  **Gender:** 105 males,  81 females  **Other specific:** University students in Shanghai, China | Smartphone | **Design:**  Experimental laboratory study  **Conditions:**   - Holding and looking at a smartphone while standing vs - Normal standing (without holding or looking at any device)   **Task:**  No task given on phone | 1. **Type of exposures:**   Gaze angle, head and neck posture  **Measurement method:**  Photogrammetry (measurements on lateral photographs)  **Variable(s):**   - Head flexion WRT vertical - Neck flexion WRT horizontal - Forward head shift - Gaze angle WRT horizontal | - No differences in head flexion between males and females during normal standing were found; but significantly higher in males (97.8° (10.5)) than in females (91.9° (11.6)) while looking at smartphone in standing than in normal standing - Significantly lower head flexion during normal standing (73.6° (9.0)) than when looking at smartphone in standing (95.2° (11.3)) were found - Neck flexion was significantly lower in males than in females during both normal standing (53.8° (6.4) vs 55.8° (5.9)) and looking at smartphone in standing (35.9° (9.2) vs 42.4° (9.2)) - Neck flexion was significantly higher during normal standing (54.7° (6.2)) than when looking at a smartphone during standing (38.8° (9.7)) - Forward head shift was significantly higher in males than in females during both normal standing (11.2° (3.5) vs 10.0° (3.6)) and when looking at a smartphone during standing (14.5° (4.7) vs 12.9° (4.5)) - Forward head shift was significantly lower during normal standing (10.9° (3.5)) than when looking at a smartphone during standing (13.9° (4.6)) - No differences in gaze angle between males and females when looking at a smartphone during standing were found |
| **Guan et al (2016) [34]** | **n** = 429  **Age:** 19.8 (2.6) years  **Gender:** 219 males,  210 females  **Other specific:** University students in Shanghai, China | Smartphone | **Design:**  Experimental laboratory study  *(a cross-sectional study was also conducted and is listed in S6)*  **Conditions:**   - Holding and looking at a smartphone in standing vs - normal standing (no holding or looking at any device)   **Task:**  No task given to perform on phone | 1. **Type of exposures:**   Gaze angle, head and neck posture  **Measurement method:**  Lateral photographs taken in front of a measuring board  **Variable(s):**   - Head and neck flexion WRT vertical - Gaze angle WRT horizontal | - When looking at a smartphone during standing, males had significantly larger head flexion (96.4° (12.2) vs. 93.6° (12.6)) and neck flexion (51.9° (9.6) vs. 47.1° (9.5)) than females - Head and neck flexion postural changes during normal standing compared to looking at a smartphone while standing were significantly larger in males compared to females - No significant correlations between head and neck postural changes and frequency of mobile phone usage in males and females were found - No differences in gaze angle between females and males were seen - No differences in normal standing head and neck postures between males and females |
| **Hong et al (2013) [22]** | **n** = 26  **Age:** 21.9 (3.3) years  **Gender:** 13 males; 13 females  **Other specific:** Students from university in South Korea | Smartphone | **Design:**  Experimental laboratory study  **Conditions:**  Typing standard text with:   - One-handed hold on smartphone vs - Two-handed hold on smartphone vs - Laptop keyboard   **Task:**  Typing | 1. **Type of exposures:**   Wrist, fingers and thumb muscle activity  **Measurement method:**  EMG on right and left ECR, FCR, APL and ED  **Variable(s):**  Mean muscle activity of:   - Right ECR, FCR (wrist) - Right ED, APL   (fingers and thumb)   - Left ECR, FCR, ED, APL   (wrist, fingers and thumb) | - Significantly lower during smartphone than during laptop keyboard typing, and in two handed than in one handed hold on smartphone - No significant differences between smartphone and laptop keyboard typing; significantly lower in two handed hold than in one handed hold on smartphone - Significantly lower during smartphone than during laptop keyboard typing, and in one handed than in two handed hold on smartphone |
| **Jacquier-Bret et al (2014) [42]** | **n** = 10  **Age:** -  **Gender:** -  **Other specific:** Right handed and novice tablet users | Tablet computer  (10” screen) | **Design:**  Experimental laboratory study  **Conditions:**  Tablet computer use with:   - Stylus vs - Fingers   **Task:**  Perform puzzle of 9 vs 16 pieces | 1. **Type of exposures:**   Elbow and wrist posture  **Measurement method:**  Motion capture system  **Variable(s):**   - Elbow and wrist flexion/extension ROM - Elbow/wrist interaction strategy (determined from slope of 95% confidence eclipse plot of wrist flexion/extension against elbow flexion/extension) | - Both elbow and wrist flexion/extension ROM for the 16 pieces puzzle were significantly higher compared to that for the 9 pieces puzzle - Elbow flexion/extension ROM was significantly higher than wrist flexion/extension ROM across all conditions - Wrist, elbow and mixed interaction strategies were shown, and differed between subjects and conditions |
| **Kietrys et al (2015) [20]** | **n** = 20  **Age:** 21.2 (2.7) years  **Gender:** 4 males, 16 females  **Other specific:** Right handed students from a university in the USA | Smartphone (3.5” screen) and tablet computer  (7” and 9.5” screen) | **Design:**  Experimental laboratory study  **Conditions:**  Device type:   - Smartphone vs - Tablet computer vs - Physical keypad phone   Touch screen size:   - 3.5” smartphone vs - 7” tablet vs - 9.5” tablet   Handhold in sitting position:   - Two-handed hold   *(portrait orientation)* vs   - One-handed hold (right hand; *portrait orientation)* vs - Preferred handhold *(portrait/landscape)*   **Task:**  Typing | 1. **Type of exposures:**   Neck and wrist posture  **Measurement method:**  Video analysis  **Variable(s):**   - Head/neck flexion - Wrist extension and ulnar/radial deviation  1. **Type of exposures:**   Neck/shoulder, wrist, fingers and thumb muscle activity  **Measurement method:**  EMG on UT, ECR, FDS, APB  **Variable(s):**  50^th^ percentile root mean square of:   - UT (neck/shoulder) - ECR (wrist) - FDS, APB (fingers and thumb)  1. **Type of exposures:**   Preferred texting styles  **Measurement method:**  Observation by investigators on participants’ preferred style of holding device and texting  **Variable(s):**   - Preferred texting style | - Head/neck flexion significantly increased with increasing screen size during two-handed hold and preferred handhold (21.9° (8.3) with 3.5” smartphone, 21.8° (9.5) with 7” tablet, 22.6° (7.0) with 9.5” tablet) - Head/neck flexion was significantly higher during smartphone use with two-handed hold (21.5° (8.3)) than one-handed hold (19.5° (8.4)) - Wrist extension was significantly higher during smartphone use than during physical keypad phone - Wrist extension and ulnar deviation increased with increasing touch screen size during two-handed hold and preferred handhold - No differences between handholds - No differences between smartphone and physical keypad phone use, and between handholds were shown - UT muscle activity increased significantly with increasing screen size during two handed hold - ECR muscle activity was significantly lower when using a smartphone than when using a physical keypad phone - ECR muscle activity increased significantly with increasing screen size during both two-handed and preferred handholds - ECR muscle activity was significantly higher during one handed than during two handed hold - Both FDS and APB muscle activity were significantly lower when using a smartphone than when using a physical keypad phone - Mean muscle activity of the FDS (but not of the APB) increased significantly with increasing screen size during two handed hold - No differences for both FDS and APB between handholds were shown - The use of both thumbs only (without digits) was adopted by 90% of participants for 3.5” smartphone (smallest screen) - The use of right index finger with any combination of other digits was adopted by 70% of participants for 9.5” tablet, 15% of participants for 7” tablet and 0% for 3.5” smartphone - Use of device on the lap was adopted by 60% of participants for 9.5” tablet, 10% of participants for 7” tablet and 0% for 3.5” smartphone |
| **Kim et al (2014) [18]** | **n** = 19  **Age:** 24.3 (6.4) years  **Gender:** 10 males, 9 females  **Other specific:** All were experienced touch typists, 17 participants were right handed | Virtual (touch screen) keyboard on a laptop | **Design:**  Experimental laboratory study  **Conditions:**  While sitting for 10 minutes, tasks were performed on three types of keyboards:   - Virtual keyboard on a laptop vs - Physical keyboard on a laptop vs - Desktop computer keyboard   **Task:**  Typing | 1. **Type of exposures:**   Neck/shoulder and fingers muscle activity  **Measurement method:**  EMG on right UT, EDC, FDS  **Variable(s):**  10^th^, 50^th^ and 90^th^ percentile muscle activity of:   - Right UT (neck/shoulder) - Right EDC (fingers) - Right FDS (fingers)  1. **Type of exposures:**   Typing forces  **Measurement method:**  Force platform under keyboard  **Variable(s):**   - Median and peak typing and individual keystroke forces | - Muscle activity (10th percentile) was significantly higher during typing on a virtual keyboard than on a physical keyboard on laptop, but did not differ from typing on a desktop computer keyboard - Muscle activity (50th and 90th percentile) was significantly lower during typing on a virtual keyboard than on a desktop computer keyboard, no differences compared to physical keyboard on laptop - Muscle activity was significantly lower during typing on virtual keyboard than on a physical keyboard on both laptop and desktop - Median and peak typing and individual keystroke forces were significantly lower when typing on a virtual keyboard than on a physical keyboard, on both laptop and desktop computer |
| **Kim et al (2014) [47]** | **n** = 21 (data analysed for 19 only)  **Age:** 24.5 years (18 to 49 years)  **Gender:** 12 males, 9 females  **Other specific:** All were right handed experienced touch typists in the USA | Virtual (touch screen) keyboard on a notebook computer | **Design:**  Experimental laboratory study  **Conditions:**  While sitting, 2x5minute tasks were executed on 4 virtual keyboard with different key sizes:   - 13, 16, 19, and 22mm (width and height), and vertical centre-to-centre key spacing of 15, 18, 21, and 24mm   **Task**:  Typing | 1. **Type of exposures:**   Wrist posture  **Measurement method:**  Bi-axial electrogoniometers  **Variable(s):**  Median (50^th^ percentile), and ROM (5^th^ and 95^th^ percentiles) of:   - Wrist flexion/ extension and ulnar/ radial deviation  1. **Type of exposures:**   Neck/shoulder and fingers muscle activity  **Measurement method:**  EMG on right UT, FDS and EDC  **Variable(s):**  Static (10th percentile), median (50^th^ percentile) and peak (90th percentile) APDF of:   - UT (neck/ shoulder) - FDS and EDC | - In general, higher median wrist extension was associated with smaller key sizes (13 & 16 mm) compared to with larger key sizes (19 & 22mm) on a virtual keyboard - No differences in wrist radial/ulnar deviation between different key sizes were shown - Greater ROM in wrist radial/ulnar deviation (0.1° to 2.7°) and wrist flexion/ extension (1.8° to 3.5°) was required in the right hand with larger key sizes (19 & 22 mm) compared to with smaller key sizes; no ROM differences were seen between key sizes in the left hand - Smallest key size (13mm) had greater static muscle activity compared to the larger key sizes (16 & 19mm); there were no differences in median and peak muscle activity between different key sizes - There were no differences in muscle activity between different key sizes |
| **Kingston et al (2016) [48]** | **n** = 14  **Age:** 22.5 (1.6) years  **Gender:** 7 males, 7 females  **Other specific:** All were right handed | Tablet computer | **Design:**  Experimental laboratory study  **Conditions:**  While sitting with a 135° thigh-to-trunk angle on a pivoting Locus chair, tasks of 4x15minutes on a hybrid workstation were performed:   - Tablet vs desktop computer - Horizontal vs sloped 15° work surface   **Tasks:**  Reading vs  Writing vs  E-mail (5 minutes for each task) | 1. **Type of exposures:**   Shoulder, elbow and wrist postures  **Measurement method:**  Motion analysis system  **Variable(s):**  Median postures of:   - Shoulder elevation and axial rotation - Elbow flexion - Wrist ulnar deviation and pronation | - Shoulder elevation during writing task (0° (2)) was higher than during reading (–6° (3)); there were no differences between emailing and writing or reading - Shoulder axial rotation when using a tablet (89° (2)) was higher than when using a desktop (82° (2)); and was higher on sloped (17° (4)) than a horizontal work surface (10° (2)) - Elbow flexion was significantly higher on a sloped work surface during reading (77° (6)) or writing (93° (4)) than on a horizontal work surface respectively by 4° and 19° ; however during emailing, elbow flexion is 9° lesser while working on a sloped work surface than on a horizontal work surface - Median elbow flexion was higher during tablet than desktop use - Range of elbow flexion was significantly smaller during tablet (28° (5)) than desktop use (42° (4)) - When using a tablet, wrist ulnar deviation during reading (9° (3)) was significantly lower than during writing (23° (2)) and emailing (17° (2)); wrist ulnar deviation was also significantly lower when using a desktop during emailing than (14° (2)) than when writing (20° (2)) and reading (19° (3)) - On a horizontal work surface, wrist ulnar deviation was significantly lower during reading (11° (2)), than during writing (20° (2)) or emailing (17° (2)); wrist ulnar deviation was also significantly lesser on sloped work surface during emailing (14° (2)), than during writing (24° (2)) and reading (17° (2)) - Wrist pronation was significantly lower during reading (60° (6)) than during writing or emailing - Median wrist pronation was higher during tablet than during desktop use - Range of wrist pronation was significantly smaller during tablet (27° (4)) than desktop use (42° (4)) |
| **Ko et al (2016) [49]** | **n** = 27  **Age:** 28.0 (4.5) years  **Gender:** 15 males, 12 females  **Other specific:** All were right handed, regular computer and smartphone users | Smartphone | **Design:**  Experimental laboratory study  **Conditions:**  Smartphone use in portrait orientation, for 2 minutes with:   - Two-handed hold at chest level - Two-handed hold at knee level (with trunk bent forward and elbows on the thighs) - Two-handed hold at eye level - One-handed hold (right side)   **Task:**  Typing | 1. **Type of exposures:**   Neck, elbow and wrist postures  **Measurement method:**  Flexible electrogoniometers  **Variable(s):**   - Neck flexion relative to a reference posture (anatomical points not specified) - Elbow flexion - Wrist flexion  1. **Type of exposures:**   Shoulder, elbow, fingers and thumb muscle activity  **Measurement method:**  EMG on UT, biceps brachii, FDS, EDC and FPB  **Variable(s):**   - Muscle activity of UT, biceps brachii, FDS, EDC and FPB | - Neck flexion was significantly higher during two-handed hold at chest level (20.8° (1.9)) and one-handed hold (18.5° (1.6)), than during two-handed hold at knee level (0.7° (2.0)) or eye level (3.4° (0.8)) - Elbow flexion during two-handed hold at chest level (90.6° (2.6)) or one-handed hold (91.2° (2.3)) was significantly higher than during two-handed hold at knee (67.9° (3.2)) or eye level (77.7° (2.9)) - No differences in wrist flexion among all the conditions were found - One-handed hold resulted in the highest muscle activities for all muscles among all conditions - Two-handed hold at knee level resulted in the lowest muscle activities in UT, biceps brachii and EDC muscles - UT muscle activity (50th percentile APDF) during two-handed hold at eye level was significantly higher than while holding the device at knee level or chest level - FDS muscle activity (50th and 90th percentile APDF) during one-handed hold was significantly higher than during two-handed hold at chest level - In general, EDC and FPB muscle activity (50th percentile APDF) during one-handed hold were significantly higher than during two-handed hold |
| **Lee et al (2015) [52]** | **n** = 18  **Age:** 20.1 (1.5) years  **Gender:**  9 males; 9 females  **Other specific:** All have at least 1 year experience using a smartphone | Smartphone | **Design:**  Experimental laboratory study  **Conditions:**  Smartphone use for 2x2minutes for each task in:   - Sitting vs - Standing   **Task:**  Text messaging vs  web browsing vs  video watching | 1. **Type of exposures:**   Head posture  **Measurement method:**  Motion analysis system  **Variable(s):**   - Head flexion WRT vertical | - Head flexion (10^th^ and 50^th^ percentile, but not the 90^th^ percentile) was significantly higher during text messaging than during web browsing and video watching - Head flexion was significantly higher in sitting than in standing across all 3 tasks (text messaging was higher by 10-14%, web browsing by 4-6% and video watching by 23-24%) - The results of the post-hoc analysis were not reported |
| **Lin et al (2015) [54]** | **n** = 18  **Age:** males 24.8 (3.5); females 23.1 (0.9) years  **Gender:** 9 males, 9 females  **Other specific:** All were right handed | Tablet computer | **Design:**  Experimental laboratory study  **Conditions:**  Tablet use on different workstations:   - Desk (flat on desk) vs - Lap (flat on lap) vs - Bed (inclined sitting on bed, tablet on lap)   Using different virtual keyboard designs:   - Standard vs - Wide vs - Split   **Task:**  Typing | 1. **Type of exposures:**   Neck, elbow and wrist posture  **Measurement method:**  Flexible electrogoniometers  **Variable(s):**  Median (50^th^ percentile) and range of APDF of:   - Neck flexion - Wrist extension and ulnar deviation - Elbow flexion | - There were no differences in neck flexion among tablet use on a desk, lap or bed, but higher range of neck flexion was found during tablet use on a desk than on the lap or bed - There were no differences in neck flexion among keyboard designs - Median wrist extension was significantly higher with tablet was on a bed (>50°) than on the lap and on a desk; wrist extension was generally higher with a wide than with a standard or split keyboard - Median wrist ulnar deviation (the left side, but not the dominant right side) was significantly higher with tablet use on a desk than on the lap; wrist ulnar deviation was significantly lower with a split compared to a standard or wide keyboard - Median elbow flexion was significantly higher during tablet use on a bed than on a desk or lap; elbow flexion was higher with a split than with a wide keyboard |
| **Ning et al (2015) [55]** | **n** = 14  **Age:** -  **Gender:** 10 males, 4 females  **Other specific:** All had no musculoskeletal disorders, neck pain or neck injury | Smartphone and tablet computer | **Design:**  Experimental laboratory study  **Conditions:**  Device use in standing for 3x90s in each task with the conditions:   - Smartphone vs tablet - Flat on table vs handheld (holding the device in the left hand)     **Task:**  Reading vs  Typing vs  Gaming | 1. **Type of exposures:**   Neck posture  **Measurement method:**  Motion analysis system  **Variable(s):**   - Head/neck flexion  1. **Type of exposures:**   Neck muscle activity  **Measurement method:**  EMG on right and left cervical extensors  **Variable(s):**   - Muscle activity of right and left cervical extensors | - Head/neck flexion was significantly higher when using a smartphone (44.7°) than a tablet computer (43.0°) - Head/neck flexion was significantly higher when using a smartphone or tablet placed flat on a table (46.4°) than when holding it (41.4°); separate mean values for smartphone and tablet were not reported - Head/neck flexion was significantly higher when typing (45.6°), than when gaming (43.6°) and reading (42.4°) - There were no significant differences in cervical extensor muscle activity between smartphone and tablet use - Muscle activity was significantly higher for the left side when using a smartphone or tablet flat on the table than in when holding it (9.4% vs 8.8%); a similar (but non-significant) trend for right side was shown - Muscle activity was significantly higher for both right and left sides during gaming and typing, than during reading; there were no significant differences between gaming and typing |
| **Pereira et al (2013) [57]** | **n** = 30  **Age**: 30.0 (11) years  **Gender:** 15 males, 15 females  **Other specific:** All were right handed, regular users of tablet or smartphone with “small hands” | Tablet computer | **Design:**  Experimental laboratory study  **Conditions:**  Tablet use with one-handed hold (left hand) while standing for 4 minutes, in 8 different configurations with 5 independent variables:   - Tablet size (small/ middle/ large) - Orientation (landscape/ portrait) - Grip shape (flat/ ledge/ handle grip) - Surface texture (smooth/ rough) - Stylus shape (small/ large/ tapered diameter)   **Tasks:**  Typing (using only the right hand) | 1. **Type of exposures:**   Gaze angle, trunk, left shoulder and wrist posture  **Measurement method:**  Motion analysis system  **Variable(s):**   - Gaze angle WRT ear–eye line, and trunk angle relative to a reference posture (anatomical points not specified) - Left shoulder moment - Left wrist extension and ulnar deviation  1. **Type of exposures:**   Neck/shoulder, wrist and fingers muscle activity  **Measurement method:**  EMG on left UT, FCR, ECR, ED, FDS; right ECR and FPB when using stylus  **Variable(s):**  50^th^ percentile APDF of:   - Left UT (neck/shoulder) - Left FCR, ECR (wrist) - Left ED, FDS (fingers) - Right ECR, FPB (wrist and thumb) | - There were no significant differences in gaze angle across all the configurations - Shoulder moment significantly increases when using a large tablet compared to a medium or small tablet - Shoulder moment significantly increases when holding a table with a flat grip (holding tablet by itself), compared to a tablet with a ledge handle and handle grip - There were no differences among different orientation and surface texture - Wrist extension significantly decreases when using a small (12.7° (29.5)) compared to a medium (19.3° (30.4)) and a large tablet (21.6° (29.2)); There were no differences for ulnar deviation - There was a significant decrease in wrist extension when using a tablet in portrait orientation (13.4 (25.7)) compared to in landscape orientation (19.6 (32.8)); There were no differences for ulnar deviation - There were no differences in wrist extension and ulnar deviation among different grip shapes and surface textures - Left UT activity significantly decreased when using a small tablet compared to when using a large tablet - There were no significant differences for orientation, grip shape and surface texture - There was a significant increase in FCR (but not for ECR) muscle activity when using a large compared to a medium and small tablet; - No significant differences for orientation, grip shape and surface texture - There was a significant increase in FDS (but not for ED) muscle activity when using a large compared to a medium or small tablet; - No significant differences for orientation, grip shape and surface texture - There were no significant differences when using different stylus shapes |
| **Shin and Kim (2014) [59]** | **n** = 15  **Age**: 26.1 (5.7) years  **Gender**: -  **Other specific:** All had no neck pain or spinal injuries in the past year | Smartphone | **Design:**  Experimental laboratory study  **Conditions:**  Smartphone use with two-handed hold for 15 minutes while sitting with:   - A neutral neck posture, with the smartphone on a desk vs - A flexed neck posture, with the smartphone on the lap   **Task:**  Free use of applications of choice | 1. **Type of exposures:**   Neck muscle activity  **Measurement method:**  EMG of CES  **Variable(s):**   - Flexion relaxation ratio *(calculated by dividing maximum muscle activity during cervical extension with return to starting position for 5s, by the average activation during sustained cervical full flexion for 5s)* | - No significant differences were found between using smartphone on a desk and on the lap - No significant differences were found before and after using smartphone on a desk or on the lap |
| **Stoffregen et al (2014) [60]** | **n** = 36  **Age:** 21.3 (2.7) years  **Gender:** 14 males, 22 females  **Other specific:** Undergraduate students from a university in the USA | Tablet computer | **Design:**  Experimental laboratory study  **Conditions:**  While sitting till motion sickness sets in or after 50 minutes tasks were performed with:   - Tablet computer supported and tilted at 45° *(touch condition)* - Two-handed hold without support, while tilting the tablet manually *(tilt condition)*   **Task:**  Gaming (finger control game) | 1. **Type of exposures:**   Head and trunk movement  **Measurement method:**  Magnetic tracking system for movement in medio-lateral and anterior-posterior direction  **Variable(s):**  Movement variability *(standard deviation of position)* and movement dynamics *(using detrended fluctuation analysis)* of:   - Head - Trunk | - There was a significant increase in movement variability of the head in medio-lateral and anterior-posterior direction over time; there were no differences between touch and tilt conditions - Movement dynamics of the head in medio-lateral and anterior-posterior direction increased over time; only in the anterior-posterior direction, dynamics was higher in touch condition than in tilt condition - There was a significant increase in movement variability of the trunk in medio-lateral direction over time; there were no differences between touch and tilt conditions - There were no differences in movement dynamics of the trunk between touch and tilt conditions |
| **Straker et al (2008) [19]** | **n** = 18  **Age:** 5.8 (0.62) years  **Gender:** 9 males, 9 females  **Other specific:** All were right handed and used a computer regularly | Tablet computer | **Design:**  Experimental laboratory study  **Conditions:**  While sitting at a desk, tasks (5 minutes) were executed using:   - Tablet computer (using stylus) vs - Desktop vs - Paper   **Task:**  Colouring-in task | 1. **Type of exposures:**   Head, neck, shoulder, scapula posture and posture variability  **Measurement method:**  Motion analysis system  **Variable(s):**   - Head flexion WRT vertical - Neck flexion WRT vertical - Cranio-cervical and cervico-thoracic angles - Trunk flexion - Head relative to trunk - Scapula elevation - Shoulder flexion - Posture variability (calculated via APDF and EVA)  1. **Type of exposures:**   Neck/shoulder muscle activity  **Measurement method:**  EMG on right and left CES, UT  **Variable(s):**   - CES and UT mean muscle activity - Muscle activity variability (calculated via APDF and EVA) | - There was significantly higher head flexion when using a tablet (110.5° (2.8)) than a desktop (85.9° (1.6)), there were no differences than paper - There was significantly higher neck flexion when using a tablet (76.3° (3.1)) than a desktop (61.5° (2.0)), there were no differences than paper - There were significantly lower cranio-cervical and cervico-thoracic angles when using a tablet than a desktop; no differences were found between tablet and paper - There were no significant differences in trunk flexion between using a tablet, desktop or paper - There was significantly higher head relative to trunk angle when using a tablet than a paper - There was significantly higher left scapula elevation when using a tablet than when using a desktop and paper, there were no differences for right side elevation - There was significantly higher right shoulder flexion when using a tablet than when using a desktop; there were no differences for left shoulder flexion - There was significantly greater posture variability when using a tablet compared to a desktop computer for head flexion, neck flexion, cranio-cervical angle, cervico-thoracic angle, as well as right and left shoulder protraction and flexion - Significantly greater posture variability in head relative to trunk position when using a paper compared to a tablet - There were significantly higher muscle activity in right CES and right UT when using a tablet compared to a desktop - There were no significant differences in left CES and left UT between tablet and paper use - There was significantly higher muscle activity variability in left CES and right UT when using a tablet compared to a desktop - There was significantly lower muscle activity variability in left UT when using a tablet compared to paper; there were no differences for right UT and CES |
| **Trudeau et al (2012) [61]** | **n** = 10  **Age:** 27.0 (7.0) years  **Gender:** 5 males, 5 females  **Other specific:** All participants were right handed | Smartphone | **Design:**  Experimental laboratory study  **Conditions:**  One-handed hold on smartphone and tapping with thumb between 2 of 12 emulated keys on different locations on the whole screen | 1. **Type of exposures:**   Wrist and thumb posture  **Measurement method:**  Motion analysis system  **Variable(s):**   - Wrist flexion/extension, ulnar/radial deviation, thumb CMC and MCP flexion/extension, abduction/adduction and IP flexion/extension | - There were significant wrist and thumb postural differences among different key locations on the screen - During tapping on the *bottom right corner of screen*, the wrist was flexed and ulnar deviated, the CMC joint was flexed and pronated, the IP and MCP joints were most flexed than in all other key locations - During tapping on the *top left corner of screen*, the wrist was extended and ulnar deviated, the CMC joint was extended and supinated, the MCP joint was extended, and the IP joint was less flexed than in all other key locations - Wrist and thumb flexion/extension differences were greatest between the *top left and bottom right corners,* except for the CMC joint - Thumb CMC and MCP abduction differences were greatest between the *top right and bottom left corners*, with greatest CMC abduction and least MCP adduction associated with the bottom left corner |
| **Trudeau et al (2013) [62]** | **n** = 12  **Age:** 30.0 (5.1) years  **Gender:** 6 males, 6 females  **Other specific:** All were right handed | Tablet computer | **Design:**  Experimental laboratory study  **Conditions:**  Tablet use with two handed hold while sitting performing tasks (for 2 minutes), for 11 configurations of 3 independent variables:   - Tablet orientation (portrait/ landscape) - Keyboard layouts (standard/ split) - Keyboard locations (top/ middle/ bottom)   **Task:**  Typing | 1. **Type of exposures:**   Wrist and thumb posture  **Measurement method:**  Motion analysis system  **Variable(s):**  Median joint angle and ROM (differences between 90^th^ and 10^th^ percentile) of:   - Wrist extension and ulnar deviation - Wrist flexion/extension, radial/ulnar deviation ROM - Thumb joint angles *(CMC extension, abduction, pronation; MCP extension, abduction; IP flexion)* - Thumb ROM *(CMC flexion/extension, abduction/adduction, supination/pronation, MCP flexion/extension, abduction/adduction, IP flexion/extension)* | - Significantly lower wrist ulnar deviation with tablet in portrait than in landscape orientation; there were no significant differences for wrist extension - Significantly lower wrist extension and ulnar deviation for split keyboard than standard keyboard layout - Significantly lower wrist ulnar deviation for bottom keyboard location, than middle and top; there was significantly lesser wrist extension for top keyboard location than middle - Significantly lower wrist flexion/ extension ROM in portrait than in landscape orientation; there were no significant differences for radial/ulnar deviation - ROM in both flexion/extension and radial/ulnar deviation significantly higher when using a standard keyboard compared to a split keyboard layout - There were no significant differences between different keyboard locations - Significantly higher IP flexion in portrait than in landscape orientation - Significantly higher CMC abduction and MCP extension when using a standard keyboard compared to a split keyboard layout - Significantly lower CMC extension, but higher CMC abduction and pronation, and MCP abduction, for bottom compared to top keyboard location - Significantly higher CMC abduction/adduction in portrait than in landscape orientation - Significantly higher CMC flexion/extension, MCP flexion/extension and abduction/adduction, and IP flexion while using a standard compared to a split keyboard layout - Significantly higher CMC flexion/extension, supination/pronation and IP flexion/extension for bottom compared to top keyboard location |
| **Trudeau et al (2016) [63]** | **n** = 10  **Age:** 27.0 (7.0) years  **Gender:** 5 males, 5 females  **Other specific:** All were right handed | Smartphone | **Design:**  Experimental laboratory study  **Conditions:**  Smartphone use with:   - One-handed hold (phone in portrait orientation) vs - Two-handed hold (landscape orientation)   **Task:**  Reciprocal tapping between 2 of 12 emulated keys on screen | 1. **Type of exposures:**   Wrist and thumb posture  **Measurement method:**  Motion analysis system  **Variable(s):**   - Wrist extension and ulnar deviation - Thumb CMC and MCP joint - Thumb IP flexion | - Wrist extension (15° (3)) was significantly higher with two handed than with one handed hold (10° (3)); there were no differences in wrist ulnar deviation between the two holds - Thumb CMC joint was significantly more extended (5° (3) vs 0° (3)), abducted (27° (2) vs 24° (1)), and supinated (0° (7) vs -8° (7)) with two handed than with one handed hold - Thumb MCP joint was significantly more extended with two handed (6° (3)) than one handed hold (4° (3)); there were no differences in MCP abduction - There were no differences in thumb IP flexion between the two holds |
| **Vasavada et al (2015) [64]** | **n** = 33  **Age:** 19 to 46 years  **Gender:** 17 males, 16 females  **Other specific:** Participants were recruited from a university in USA and used tablet for ≥1 month | Tablet computer | **Design:**  Experimental laboratory study  **Conditions:**  Tablet was used in landscape orientation while sitting, executing tasks (2 to 5 minutes) in:   - Tilted high (73°) on desk - Tilted low (15°) on desk - Flat on desk - Tilted low (15°) on lap - Self-selected position   **Task:**  Reading or typing | 1. **Type of exposures:**   Head, neck and trunk posture  **Measurement method:**  Analysis of photographs taken  **Variable(s):**   - Head flexion WRT horizontal - Neck flexion WRT horizontal - Trunk flexion - Head-neck and neck-trunk angles  1. **Type of exposures:**   Gravitational demand at head and neck  **Measurement method:**  Modelling done from photographs and radiographs taken  **Variable(s):**   - Gravitational demand at C6-C7 centre of rotation *(ratio of gravitational moment and muscle moment)* | - Significantly less flexed during reading with a tablet tilted at a high angle on a desk (-5.1° (8.2)), than when flat on a desk (-13.6° (8.2)) and tilted low on the lap (-21.7° (9.4)) - Significantly less flexed during both reading and typing with tablet tilted low on a desk, than flat on desk and tilted low on the lap - Significantly more flexed during reading with the tablet flat on a desk (100.5° (7.8)) than while tilted high on desk (95.7° (8.0)) - Head/neck and neck/trunk angles were significantly higher during reading with tablet flat on the desk and tilted low on lap, than tilted high on a desk - Head/neck and neck/trunk angles were significantly higher during reading and typing with the tablet tilted low on the lap, than when tilted low on desk - Gravitational demands were significantly higher in all the tablet conditions compared to neutral head/neck posture - Gravitational demands were significantly higher during reading with tablet flat on desk than tilted high on desk; no significant differences among other conditions - Significantly higher during typing with the tablet tilted low on the lap than tilted when low on a desk and flat on a desk - No significant differences between reading and typing tasks were found |
| **Werth & Babski-Reeves (2014) [16]** | **n** = 12  **Age:** 23.3 (2.7) years  **Gender:** 6 males, 6 females  **Other specific:** Students from a university in the USA that were touch typists | Tablet computer | **Design:**  Experimental laboratory study  **Conditions:**  Device use while sitting performing tasks (30 minutes) on:   - Tablet computer vs   netbook vs  laptop   - Desk vs sofa   **Task:**  Typing | 1. **Type of exposures:**   Neck, elbow and wrist posture  **Measurement method:**  Electrogoniometers on neck, right and left upper and lower arms and wrists  **Variable(s):**   - Neck flexion relative to a reference posture (anatomical points not specified) - Neck rotation - Elbow flexion/extension - Wrist flexion/extension - Wrist radial/ulnar deviation  1. **Type of exposures:**   Neck/shoulder and wrist muscle activity  **Measurement method:**  EMG on SCM, UT, FCR, ECR  **Variable(s):**  Mean muscle activity of:   - UT, SCM, ECR, FCR | - Significantly higher when using device on a sofa (9.46° (0.79)) than on a desk (3.03° (8.05)) - Higher (but non-significant differences) when using tablet compared to laptop or netbook - No significant differences in neck rotation between tablet, netbook and laptop use, and between use on a desk or sofa were found - Significantly higher elbow flexion when using device on a sofa (4.31° (14.62)) compared to on a desk (1.26° (14.79)) - There were no significant differences between tablet, netbook and laptop - Significantly higher wrist extension when using a device on a sofa compared to on a desk - Significantly higher wrist extension when using a tablet (wrist extension 12.61°) compared to a laptop (wrist flexion 2.06°) - Significantly lower wrist ulnar deviation when using a tablet (10.04°) than a laptop (17.30°) - There were no significant differences between use on desk and sofa - There were significantly lower muscle activity for all the tested muscles when using a tablet, compared to a netbook and laptop - Post-hoc analysis was unable to identify specific differences between activity of the different muscles and the conditions |
| **Xiong and Muraki (2014) [65]** | **n** = 20  **Age:** 24.5 (2.2) years  **Gender:** 10 males, 10 females  **Other specific:** Participants were right handed students from a university in Japan | Smartphone | **Design:**  Experimental laboratory study  **Conditions:**  Mock up smartphone use on desk, performing standardized tasks at:   - Fixed vs max speed   **Task:**  Tapping *(large vs small buttons)*  Moving *(abduction-adduction vs flexion-extension orientations*)  Circling *(clockwise vs counter-clockwise directions)* | 1. **Type of exposures:**   Thumb muscle activity  **Measurement method:**  EMG on APB, FDI, FPB and ED *(FPB and ED results not reported by authors as no significant findings were found)*  **Variable(s):**  Integrated EMG (iEMG), muscle contraction time and iEMG divided by contraction time (iEMG/s) of:   - APB - FDI | - For *tapping task* from large buttons to small buttons, iEMG and contraction time of APB increased significantly at both fixed and max speed; while iEMG/s of APB only increased significantly at max speed but not at fixed speed - For *moving task* from adduction-abduction to flexion-extension task, iEMG, iEMG/s and contraction time of APB decreased significantly in both fixed and max speed - For *circling task***,** no significant differences between clockwise and counter-clockwise, and between fixed and max speed - For *tapping task* from large buttons to small buttons, iEMG and contraction time of FDI increased significantly at both fixed and max speed; while iEMG/s of FDI only increased significantly at max speed but not at fixed speed - For *moving task* from adduction-abduction to flexion-extension task, iEMG, iEMG/s and contraction time of FDI increased significantly in both fixed and max speed - For *circling task,* no significant differences between clockwise and counter-clockwise and between fixed and max speed |
| **Young et al (2012) [67]** | **n** = 15  **Age:** 29 (5.0) years  **Gender:** 7 males, 8 females  **Other specific:** All had experience with using tablet | Tablet computer | **Design:**  Experimental laboratory study  **Conditions:**  Tablet use in landscape orientation, sitting on lounge chair:   - Smaller tablet (smaller screen size and lighter) vs - Larger tablet (larger screen size and heavier)   4 configurations of varied tablet location and support:   - Lap-Hand *(on lap, one and two handed with self-selected tilt)* - Lap-Case *(on lap with lower case tilt - 15^0^ for smaller tablet; 45^0^ for larger tablet)* - Table-Case *(on desk with lower case tilt - 15^0^ for smaller tablet; 45^0^ for larger tablet)* - Table-Movie *(on desk with higher case tilt - 73^0^ for smaller tablet; 63^0^ for larger tablet)*   **Task:**  Movie watching *(only for Table-Movie)*  Gaming *(only for Lap-Hand)*  Internet browsing, reading or typing email *(for Lap-Hand, Lap-Case, Table-Case)* | 1. **Type of exposures:**   Head and neck posture  **Measurement method:**  Motion analysis system  **Variable(s):**   - Head flexion WRT vertical - Neck flexion WRT vertical - Cranio-cervical angle | - Significantly higher when using a smaller tablet (98° (2)) than a larger tablet (95° (2)) (placed at different tilt angles) - Significantly lower when using a tablet in Tablet-Movie (85° (2)) compared to all the other conditions - No significant differences between Lap-Case, Table-Case and Lap-Hand conditions - Significantly higher when using a smaller tablet (50° (2)) than a larger tablet (47° (2)) (placed at different tilt angles) - Significantly lower when using a tablet in Lap-Hand (49° (2)) than a Lap-Case (52° (2)) and Table-Case (54° (2)), but higher than Table-Movie (40° (2)) conditions - Significantly lower when using tablet in Table-Movie than in Lap-Hand, Lap-Case and Table-Case conditions - No significant differences between Lap-Case and Table-Case - No significant differences between smaller and larger tablet (placed at different tilt angles) - Significantly lower when using tablet in Lap-Hand (129° (2)) and in Lap-Case (130° (2)), than in Table-Case (135° (2)) or Table-Movie (135° (2)) conditions - No significant differences between Lap-Hand and Lap-Case, and between Table-Case and Table-Movie |
| **Young et al (2013) [68]** | **n** = 15  **Age:** 29 (5.0) years  **Gender:** 7 males, 8 females  **Other specific:** All had experience with using tablet | Tablet computer | **Design:**  Experimental laboratory study  **Conditions:**  Tablet use in landscape orientation, sitting on lounge chair:   - Smaller tablet (smaller screen size and lighter) - Larger tablet (larger screen size and heavier)   7 configurations of varied tablet location, support and task:  *No case tilt for:*   - One-handed gaming - One-handed internet browsing - Two-handed internet browsing   *With case tilt for tablet:*   - On lap typing email - On lap internet browsing - On desk typing email - On desk internet browsing   **Task:**  Gaming (for 3 minutes) vs Typing email (for 3 minutes) vs  Internet browsing (for 5 minutes) | 1. **Type of exposures:**   Shoulder and wrist posture **Measurement method:**  Motion analysis system  **Variable(s):**   - Shoulder flexion, abduction and elevation - Wrist extension - Wrist ulnar/radial deviation and acceleration  1. **Type of exposures:**   Shoulder and wrist muscle activity  **Measurement method:**  EMG on anterior deltoid, UT, FCR, FCU and ECU/ED  **Variable(s):**  10th, 50th and 90th percentile muscle activity of:   - UT (neck/shoulder) - Anterior deltoid (shoulder) - ECU/ED (wrist) - FCR and FCU (wrist) | - Highest shoulder flexion (24° (2)), lowest shoulder abduction (7° (1)), highest shoulder elevation (6° (2)) when using the tablet on desk typing email - Significantly higher shoulder flexion and elevation when using tablet for typing email on a desk than on the lap - There were no significant differences between one handed gaming and one and two handed internet browsing - Highest wrist extension when using tablet on lap typing email (35° (2)) or internet browsing (30° (2)) were found compared to on a tablet or one or two handed use - There were no significant differences between smaller and larger tablet - There was significantly higher wrist ulnar deviation when typing email (7° (2)) than the other conditions - Highest wrist ulnar/radial deviation acceleration was found when typing email on the lap (179°/s^2^ (13)) and on a table (179°/s^2^ (13)) - Significantly higher (*10^th^ and 50^th^ percentile only)* activity was found during one compared to two handed gaming and internet browsing - Significantly higher when using a tablet on a desk than on the lap typing email and on the lap internet browsing - Significantly higher *(90^th^ percentile only)* when using tablet on desk than on the lap for internet browsing - Significantly higher when using the tablet on the desk, typing email than on desk internet browsing - Significantly higher at wrist extensors when typing email than internet browsing on tablet (for both on desk and on lap) - Significantly higher *(50^th^ percentile only)* during one handed than two handed internet browsing on tablet - Muscle activity in FCR and FCU were generally low across all the configurations - Significantly higher *(90^th^ percentile only)* when typing email than internet browsing |

***Abbreviated terms:*** *ADM: abductor digiti minimi; AP: adductor pollicis; APB: abductor pollicis brevis; APDF: amplitude probability distribution function; APL: abductor pollicis longus; CES: cervical erector spinae; CMC: carpometacarpal; ECR: extensor carpi radialis; ED: extensor digitorium; EDC: extensor digitorum communis; EMG: electromyography; EVA : exposure variation analysis; FCR: flexor carpi radialis; FDI: first dorsal interosseous; FDS: flexor digitorium superficialis; FPB: flexor pollicis brevis; FPL: flexor pollicis longus; IP: interphalangeal; MCP: metacarpophalangeal; OP: opponens pollicis; ROM: range of motion; SCM: sternocleidomastoid; UT: upper trapezius; WRT: with respect to*
