# Supplementary material for: The associations of mobile touch screen device use with musculoskeletal symptoms and exposures: A systematic review
Source: PLoS One. 2017 Aug 7;12(8):e0181220. doi: 10.1371/journal.pone.0181220 (PMC5546699; doi:10.1371/journal.pone.0181220)
Supplement: S9 File — (DOCX) [file pone.0181220.s009.docx]

**S9. Summary of included case-control laboratory studies (MTSD use and physiological responses)**

| **Author** | **Study population** | **Type of MTSD examined** | **Study design and conditions** | **Physiological responses measurement** | **Physiological responses**  **results** |
| --- | --- | --- | --- | --- | --- |
| **Inal et al (2015) [41]** | **n** = 102  **Age:** 18 to 23 years  **Gender:** 30 males, 72 females  **Other specific:** University students from Turkey (66 had a habit of one-handed smartphone use) | Smartphone | **Design:**  Case-control laboratory study  Based on smartphone addiction scale, participants were grouped into (using the median score of 84 to classify high and low users):   - High users (≥84 score), vs - Low users (≤84 score) vs - Non-users   **Conditions:** NA | **1) Type of physiological responses:**  Hand function  **Measurement method:**  Duruöz Hand Index  **Variable(s):**   - Duruöz Hand Index median scores  1. **Type of physiological responses:**   Grip strength and pinch strength  **Measurement method:**  Jamar hand dynamometer and pinch meter  **Variable(s):**   - Grip strength and pinch strength median readings  1. **Type of physiological responses:**   FPL (flexor pollicis longus) and median nerve cross-sectional area and ratio  **Measurement method:**  Ultrasound over bilateral median nerve at wrist joint, and FPL at mid thenar (FPL-MT) and metacarpophalangeal joint (FPL-MCP)  **Variable(s):**   - FPL and median nerve cross-sectional area - Median nerve, FPL-MT and FPL-MCP ratio | - No differences in hand functioning among non-smartphone users, low and high smartphone users were seen - Hand functioning correlated significantly with smartphone addiction scale scores (r=0.245) - No differences in grip and pinch strength were seen among non-smartphone users, low and high smartphone users - Pinch strength, but not grip strength, correlated significantly with smartphone addiction scale scores (r=-0.281) - FPL cross-sectional area was significantly larger in the dominant arm side compared to the non-dominant side for all the 3 groups - Median nerve cross-sectional area in the dominant arm side was significantly larger among high smartphone users compared to the non-dominant arm side; no differences between dominant and non-dominant arm were seen in low or non-smartphone users - FPL-MCP cross-sectional area correlated significantly positive with the duration of smartphone use - Median nerve ratio in non-users was significantly higher compared to in the high users group, but did not differ from the low users group; higher values for the dominant hand compared to non-dominant hand in the high user group, but not in the other groups were seen. - For FPL–MT ratio, no between group differences, but higher values for the dominant hand compared to non-dominant hand in each of the groups were seen - For FPL–MCP ratio, significant group differences across all the groups; higher values for the dominant hand compared to non-dominant hand in each of the groups were seen - FPL-MT and median nerve ratio correlated significantly positive with the duration of smartphone use |
| **Jung et al (2016) [43]** | **n** = 50  **Age:** 21.0 (2.4) years  **Gender:** -  **Other specific:**  Recruited from a university in South Korea | Smartphone | **Design:**  Case-control laboratory study  Categorised into 2 groups according to reported smartphone usage   - Low users   (n=50, <4 hours/day)   - High users   (n=50, >4 hours/day)  **Conditions:** NA | 1. **Type of physiological responses:**   Respiratory function  **Measurement method:**  Spirometer  **Variable(s):**   - Peak expiratory flow (PEF) - Forced vital capacity (FVC), forced expiratory volume in 1 second (FEV1), ratio of forced expiratory volume in 1 second to forced vital capacity (FEV1/FVC) | - PEF was significantly lower in high smartphone users (4.3 (1.5)) compared to in low users (6.2 (2.3)) - No differences for FVC, FEV1 and FEV1/FVC were seen between high and low users |
| **Lee and Seo (2014) [50]** | **n** =30 (selected out of 300 surveyed)  **Age:** normal group 22.6 (1.3) years;  moderate addiction 21.5 (1.9) years; severe addiction  22.4 (2.0) years  **Gender:** 12 males, 18 females  **Other specific:** Students from a university in South Korea | Smartphone | **Design:**  Case-control laboratory study  Based on smartphone addiction scale scores, grouped into:   - Normal group (n=10; scores <40) - Moderate addiction (n=10; scores 40-43) - Severe addiction (n=10; scores >43)   **Conditions:** NA | **1) Type of physiological responses:**  Cervical repositioning error  **Measurement method:**  ROM meter applied on head to measure repositioning error compared to target position of 30^0^ cervical flexion from neutral cervical position  **Variable(s):**  Mean repositioning error for:   - Cervical flexion - Cervical extension, right and left cervical lateral flexion | - Error in severe addiction group (3.2^0^ (0.8)) was significantly higher than in the normal group (1.0^0^ (0.2)), no differences with moderate group (1.7^0^ (0.4)) were seen - Error in cervical extension, right and left cervical lateral flexion was higher in the moderate addiction compared to the normal group, and also the in severe addiction group compared to the normal group |
| **Lee et al (2012) [51]** | **n** = 125  **Age:** 21.4 (2.0) years  **Gender:** 32 males, 93 females  **Other specific:** Students from a university in South Korea, and no wrist symptoms or injuries | Smartphone | **Design:**  Case-control laboratory study  Based on a nomophobia syndrome questionnaire, participants were grouped into 3 groups for each of the categories:   - Smartphone addiction degree - Daily usage duration - Continuous using time - Total periods of use   **Conditions:** NA | **1) Type of physiological responses:**  Median nerve thickness  **Measurement method:**  Ultrasonography  **Variable(s):**   - Mean median nerve thickness | - No significant differences between median nerve thickness and smartphone addiction degree (≤19, 20-29 or ≥30 points), daily usage duration (≤3, 3-7 or ≥8 hours), continuous using time (≤30, <60 or ≥60 mins) and total periods of use (≤12, 13-24 or ≥25 months) on median nerve thickness were seen |
| **Park et al (2015) [56]** | **n =** 20  **Age:** 23.3 (2.3) years  **Gender:** -  **Other specific:** Participants were recruited from a university in South Korea | Smartphone | **Design:**  Case-control laboratory study  **Conditions:**  According to scores on the smartphone addiction scale, grouped into:   - Heavy user group (n=10, scores 45.1 (3.6)) vs - Control group (n=10, scores 26.5 (4.5)) | 1. **Type of physiological responses:**   Pressure pain threshold  **Measurement method:**  Electronic algometer, pressure applied over trigger points in SCM (sternocleidomastoid) and UT (upper trapezius)  **Variable(s):**   - Pressure pain threshold | - Significantly lower in heavy user group than in the control group |
| **Xie et al (2016) [21]** | **n** = 40  **Age:** 23.9 (3.2) years  **Gender:** 16 males, 24 females  **Other specific:** Participants were right handed and recruited from universities in Hong Kong | Smartphone | **Design:**  Case-control laboratory study  Based on questionnaires responses, participants were grouped into:   - Case group (n=20, with neck/shoulder discomfort) vs - Control group (n=20, no neck/shoulder discomfort)   **Conditions:**  While sitting for 10 minutes:   - Two-handed texting (both thumbs) at chest level vs - One-handed texting (right thumb) at chest level vs - Two-handed typing on desktop computer   **Task:**  Typing | 1. **Type of physiological responses:**   Perceived exertion  **Measurement method:**  Borg scale  **Variable(s):**   - Rating of perceived exertion | - Exertion was significantly higher in the case group than in the control group after either one or two handed smartphone texting or desktop typing - There were no differences among smartphone texting and desktop typing tasks within each group |
