# Supplementary material for: The associations of mobile touch screen device use with musculoskeletal symptoms and exposures: A systematic review
Source: PLoS One. 2017 Aug 7;12(8):e0181220. doi: 10.1371/journal.pone.0181220 (PMC5546699; doi:10.1371/journal.pone.0181220)
Supplement: S10 File — (DOCX) [file pone.0181220.s010.docx]

**S10. Summary of included experimental laboratory studies (MTSD use and physiological responses)**

| **Author** | **Study population** | **Type of MTSD examined** | **Study design and conditions** | **Physiological responses measurement** | **Physiological responses**  **results** |
| --- | --- | --- | --- | --- | --- |
| **Choi et al (2016) [39]** | **n** = 15  **Age:** 23.6 (2.4) years  **Gender:** 8 males,  7 females  **Other specific:** College students in South Korea | Smartphone | **Design:**  Experimental laboratory study  **Conditions:**  Continuous texting while sitting for 5 minutes with:   - Neutral neck *(upper part of phone screen at eye level)* - Middle neck bending *(neck bent in comfortable manner)* - Maximum neck bending   **Task:**  Typing | 1. **Type of physiological responses:**   Neck/shoulder muscle fatigue  **Measurement method:**  EMG on right and left splenius capitis and UT (without information on how values for fatigue were calculated or obtained)  **Variable(s):**   - Mean muscle fatigue | - Significantly higher muscle fatigue was seen for right and left splenius capitis, and left UT during smartphone texting in a maximum compared to a middle neck bending posture; no differences between neutral and middle/ maximum neck bending postures were seen |
| **Kim et al (2012) [46]** | **n** = 40  **Age:** 20-27 years  **Gender:** 17 males, 23 females | Smartphone | **Design:**  Experimental laboratory study  **Conditions:**  While sitting for 10 minutes two-handed use on:   - Smartphone (n=15) vs - Computer keyboard (n=15) vs - Control group (n=10)   **Task:**  Typing | **1) Type of physiological responses:**  Pressure pain threshold  **Measurement method:**  Digital pressure algometer on UT  **Variable(s):**   - Threshold force required for subjects to report “slight pain”   **2) Type of physiological response:**  Neck/shoulder, elbow, wrist and thumb muscle fatigue  **Measurement method:**  EMG on UT, brachioradialis, FCU, APB  **Variable(s):**  Median frequency of EMG of:   - UT (neck/shoulder) - Brachioradialis (elbow) - FCU and APB (wrist and thumb) | - Significantly lower pressure pain threshold after typing on smartphone (55.72 kg/cm^2^) than before typing on smartphone (64.23 kg/cm^2^) were seen - Differences between smartphone and computer keyboard typing were not reported - No significant differences between smartphone and computer keyboard typing were found - Significantly higher in the smartphone than in the control group; no significant differences between smartphone and computer keyboard were shown - Significantly lower after typing on a smartphone than before typing on a smartphone - No significant differences between smartphone and computer keyboard typing, and between before and after smartphone typing were seen |
| **Pereira et al (2013) [57]** | **n** = 30  **Age**: 30.0 (11) years  **Gender:** 15 males, 15 females  **Other specific:** Participants were right handed, regular users of tablet or smartphone and had “small hands” | Tablet computer | **Design:**  Experimental laboratory study  **Conditions:**  Tablet use with one-handed hold (left hand) while standing for 4 minutes, in 8 different configurations varying 5 independent variables:   - Tablet size (small/ middle/ large) - Orientation (landscape/ portrait) - Grip shape (flat/ ledge/ handle grip) - Surface texture (smooth/ rough) - Stylus shape (small/ large/ tapered diameter)   **Tasks:**  Typing (using only right hand) | 1. **Type of physiological responses:**   Fatigue in the neck, shoulder, forearm and wrist  **Measurement method:**  Numeric scale 1 (highest) to 7 (lowest)  **Variable(s):**   - Mean score of fatigue in neck, shoulder, forearm and wrist | - Significant increase in fatigue for all areas with increasing size of tablet were shown, and also for flat grip compared to ledge and handle grip - No differences among different orientation and surface textures were shown - Significant higher hand/wrist fatigue when using small stylus compared to tapered stylus |
| **Shim (2012) [58]** | **n** = 20  **Age:** 22.3 (0.8) years  **Gender:** -  **Other specific:** All had no neck/shoulder and upper extremity symptoms | Smartphone | **Design:**  Experimental laboratory study  **Conditions:**  Smartphone use while sitting for 30 minutes  **Task:**  Not reported | 1. **Type of physiological responses:**   Median nerve size  **Measurement method:**  Ultrasonography  **Variable(s):**   - Mean median nerve circumference (cm) - Mean median nerve area (mm^2^) - Mean distance between highest and lowest point of median nerve to lunate | - Median nerve circumference was significantly lower after smartphone use (1.12 (0.08)) compared to before use (1.39 (0.08)) - Median nerve area was significantly lower after smartphone use (8.12 (1.4)) compared to before use (10.78 (0.95)) - Both were significantly higher after smartphone use compared to before use |
| **Xiong and Muraki (2014) [65]** | **n** = 20  **Age:** 24.5 (2.2) years  **Gender:** 10 males, 10 females  **Other specific:** Right handed students from a university in Japan | Smartphone | **Design:**  Experimental laboratory study  **Conditions:**  Mock-up smartphone use on desk, perform standardized tasks at:   - Fixed vs maximum speed   **Task:**  Tapping *(large vs small buttons)*  Moving *(abduction-adduction vs flexion-extension orientations*)  Circling *(clockwise vs counter-clockwise directions)* | 1. **Type of physiological responses:**   Thumb muscle fatigue  **Measurement method:**  Time from start to end of tapping  **Variable(s):**   - Thumb muscle fatigue time  1. **Type of physiological response:**   Perceived exertion of thumb muscles  **Measurement method:**  Borg’s CR-10 scale of perceived exertion of AP, FPB, APB, FDI and ED  **Variable(s):**   - Perceived exertion score | - For *tapping task*, significantly shorter fatigue time for small buttons than large buttons, but no significant differences between fixed and maximum speed were shown - For *moving and circling task*, no significant differences in both fixed and maximum speed between abduction-adduction and flexion-extension were shown - For *tapping task*, significantly higher perceived exertion for FDI when using small buttons compared to large buttons; no significant differences for other thumb muscles - For *moving task*, significantly higher perceived exertion for APB and APL during adduction-abduction than flexion-extension; while for FDI significantly higher perceived exertion during flexion-extension than adduction-abduction - For *circling task*, no significant differences between clockwise and counter-clockwise directions were shown |

***Abbreviated terms****: AP: adductor pollicis; APB: abductor pollicis brevis; EMG; electromyography; FCU: flexor carpi ulnaris; FDI: first dorsal interosseous FPB: flexor pollicis brevis; ED: extensor digitorium; UT: upper trapezius*
